# Generation of a Zebrafish Knock-In Model Recapitulating Childhood ETV6::RUNX1-Positive B-Cell Precursor Acute Lymphoblastic Leukemia

**DOI:** 10.3390/cancers15245821

**Published:** 2023-12-13

**Authors:** Veronika Zapilko, Sanni Moisio, Mataleena Parikka, Merja Heinäniemi, Olli Lohi

**Affiliations:** 1 Tampere Center for Child, Adolescent and Maternal Health Research, Faculty of Medicine and Health Technology, Tampere University, 33100 Tampere, Finland; veronika.zapilko@tuni.fi; 2 The Institute of Biomedicine, University of Eastern Finland, 70210 Kuopio, Finland; sanni.moisio@uef.fi (S.M.); merja.heinaniemi@uef.fi (M.H.); 3 Laboratory of Infection Biology, Faculty of Medicine and Health Technology, Tampere University, 33100 Tampere, Finland; mataleena.parikka@tuni.fi; 4 Department of Pediatrics and Tays Cancer Center, Tampere University Hospital, Wellbeing Services County of Pirkanmaa, 33520 Tampere, Finland

**Keywords:** childhood pB-ALL, disease model, CRISPR/Cas9-based knock-in, zebrafish

## Abstract

**Simple Summary:**

Despite remarkable progress in the treatment of acute lymphoblastic leukemia (ALL) in recent years, it remains a significant contributor to pediatric cancer-related deaths. This highlights the urgent need for innovative therapeutic strategies that target the genetic alterations driving ALL. We built a novel zebrafish disease model for *ETV6::RUNX1*-positive ALL, which harbors secondary lesions in the two commonly mutated genes, *pax5*, and *cdkn2a/b*. The introduction of secondary mutations significantly augmented the incidence of disease. This model provides a valuable tool for investigating the etiological role of secondary mutations and facilitating the evaluation of drug sensitivities in the future.

**Abstract:**

Approximately 25% of children with B-cell precursor acute lymphoblastic leukemia (pB-ALL) harbor the *t(12;21)(p13;q22)* translocation, leading to the *ETV6::RUNX1 (E::R)* fusion gene. This translocation occurs in utero, but the disease is much less common than the prevalence of the fusion in newborns, suggesting that secondary mutations are required for overt leukemia. The role of these secondary mutations remains unclear and may contribute to treatment resistance and disease recurrence. We developed a zebrafish model for *E::R* leukemia using CRISPR/Cas9 to introduce the human *RUNX1* gene into zebrafish *etv6* intron 5, resulting in *E::R* fusion gene expression controlled by the endogenous *etv6* promoter. As seen by GFP fluorescence at a single-cell level, the model correctly expressed the fusion protein in the right places in zebrafish embryos. The *E::R* fusion expression induced an expansion of the progenitor cell pool and led to a low 2% frequency of leukemia. The introduction of targeted *pax5* and *cdkn2a/b* gene mutations, mimicking secondary mutations, in the *E::R* line significantly increased the incidence in leukemia. Transcriptomics revealed that the *E::R;pax5*mut leukemias exclusively represented B-lineage disease. This novel *E::R* zebrafish model faithfully recapitulates human disease and offers a valuable tool for a more detailed analysis of disease biology in this subtype.

## 1. Introduction

A common feature of childhood acute lymphoblastic leukemia (ALL) is the frequent occurrence of recurring chromosomal translocations [1]. The most prevalent translocation, *t(12;21)(p13;q22)*, is found in 25% of B-cell precursor ALL (pB-ALL) cases and creates an in-frame fusion that includes the repressive domain of the transcription factor ETV6 and the RUNT DNA binding domain of the transcription factor RUNX1 [2,3,4,5].

Studies involving monozygotic twins diagnosed with pB-ALL, along with dried blood spot card studies, have demonstrated that the *E::R* fusion arises in utero [6,7,8]. It occurs in ≈1–5% of normal newborns, which is far above the leukemia incidence of ≈0.01% [6,9,10]. The latency between its formation and the disease presentation can be up to a decade or more [7,11]. Moreover, studies in mice have revealed that the *E::R* fusion gene, when expressed under the control of the endogenous *Etv6* promoter, induces leukemia at a notably low frequency. A higher incidence of leukemia occurred after introducing additional genetic defects through methods such as chemical mutagenesis, *Sleeping Beauty* transposon expression, or crossbreeding with mice harboring *Pax5* or *Cdkn2a/b* deletions [12,13,14]. These observations indicate that the E::R fusion protein is insufficient for the induction of overt ALL, suggesting that secondary mutations are required for leukemogenesis. Recent studies have revealed that these mutations differ between patients [15,16].

Children with *E::R*-positive pB-ALL are known to have favorable outcomes, with event-free survival up to 97%, as reported in recent studies [17,18,19,20]. However, a subset of patients experience relapse, and conventional treatment strategies lead to significant therapy burdens and severe long-term side effects, calling for novel and improved treatment strategies [18]. Since there is evidence suggesting that secondary oncogenic mutations can predict drug responses, there is potential for the creation of personalized therapeutic strategies [21,22]. To develop such approaches, it is crucial to employ a model that possesses conserved genetic processes controlling hematopoiesis, that are easily genetically manipulable, and that has high fecundity. The zebrafish, *Danio rerio*, fulfills these criteria [23,24,25,26,27,28]. Furthermore, tumors developing in zebrafish cancer models exhibit histological and molecular similarities to their human counterparts, emphasizing their suitability for pre-clinical research [29,30,31,32,33].

Recently, an *E::R* zebrafish model expressing the human E::R fusion protein under the control of a ubiquitous promoter was developed. Around 3% of the fish developed pB-ALL, and this model was successfully utilized to identify the cellular origin of *E::R*-induced leukemia [31]. One limitation of this model was the ubiquitous expression of the E::R fusion protein, deviating from physiological sites and levels. To address this constraint, we used the CRISPR/Cas9 technology to bring the *E::R* fusion gene expression under the control of the endogenous *etv6* promoter, thus confining its expression to *bona fide etv6*-expressing cells at physiological levels. Subsequently, we modified this model by introducing targeted lesions in the *pax5* and *cdkn2a/b* genes that are commonly mutated in *E::R*-positive childhood pB-ALL.

## 2. Materials and Methods

### 2.1. Donor Plasmid Construction

To establish an *etv6*^+/*RUNX1*^ knock-in zebrafish line *(E::R* zebrafish line), we utilized the GeneWeld technique, which employs the CRISPR/Cas9 genome editing system and short homology arms for directed integration of transgenes into the genome [34,35]. To enable this, we created a donor plasmid compatible with the GeneWeld method by modifying the original *pGTag-TagRFP-B-actin* vector (Addgene, Watertown, MA, USA, #117808) [34]. First, we replaced the insert *(TagRFP-B-actin-terminator)* of the vector with the gene-breaking cassette (3440 bp), which contained the following components in the 5’ to 3’ direction: the carp b-actin intron 1 splice acceptor, a partial cDNA of human *RUNX1* (NM_001754; exon 2–8), the P2A sequence from porcine teschovirus-1, the cDNA of GAL4-VP16, and the ocean pout antifreeze gene transcriptional termination and polyadenylation (TE) sequence. Plasmid construction was achieved by sequentially inserting PCR fragments or annealed oligonucleotides (oligos). The plasmids *PME-Gal4VP16*, *pGBT-RP2-1*, and a plasmid containing *RUNX1* human untagged clone (Origene, Rockville, MD, USA; #SC123977) (Addgene, Watertown, MA, USA, #31828) served as templates for PCR reactions [36,37]. A detailed description of the vector assembly, the sequences of the oligos utilized for its construction, and the sequence of the plasmid can be found in Supplementary Materials (Method S1, Figure S1, and Table S1).

We proceeded by integrating appropriate homology arms into the newly generated plasmid. To achieve this, we first identified a target site within intron 5 of the zebrafish *etv6* gene (BC045451.1) using CRISPRScan (http://www.crisprscan.org/; accessed on the 2 February 2020) (Method S2, Figure S2, Table S2) [38]. Subsequently, 5′ and 3′ homology arms were designed, each spanning 48 base pairs and flanking the desired genomic double-strand break (DSB) site. These homology arms were incorporated into the newly formed plasmid as annealed oligos, thus framing the gene-breaking cassette. The protocol outlined by Welker et al. in 2021 was followed, and a summary of the homologous arm design is shown in Figure S3 [35]. Verification of the donor plasmid sequence was performed by Sanger sequencing and restriction digest analysis.

### 2.2. In Vitro Transcription of sgRNAs and Cas9 mRNA

Genomic target sites in exon 3 and exon 5 of *pax5* (BX511134.8) and exon 2 of *cdkn2a/b* (CT573245.20) were identified using CRISPRscan (Table S2) [38]. DNA templates with a 5’ T7 promoter followed by the target sgRNA sequence were assembled by annealing oligos and amplified using PCR as outlined in Varshney et al., 2015 (Table S3) [39]. In vitro, transcription reactions were initiated using the MEGAscript™ T7 Transcription Kit (Invitrogen, Carlsbad, CA, USA) by adhering to the manufacturer’s guidelines.

Capped Cas9 mRNA was generated through in vitro transcription using the *XbaI*-linearized plasmid *pT3TS-nCas9n* (Addgene, Watertown, MA, USA, #46757) as the template and the mMESSAGE mMACHINE T3 Kit (Invitrogen, Carlsbad, CA, USA) [40]. The kit was used according to the manufacturer’s recommendations.

The synthesized RNAs were purified using the MEGAclear™ Transcription Clean-Up Kit (Invitrogen, Carlsbad, CA, USA). To ensure quality, the concentrations of the Cas9 mRNA and the sgRNAs were measured with a Nanodrop, and their integrity was checked on a 0.9% or 1.5% agarose gel, respectively.

To evaluate sgRNA-guided Cas9 mRNA activity, we conducted a T7 endonuclease I assay (New England Biolabs, Ipswich, MA, USA) as previously described, using the primers listed in Table S4 for the amplification of the respective genomic regions [41].

### 2.3. Generation of the Transgenic and Mutant Zebrafish Lines

To establish the *E::R* zebrafish line, we microinjected 2 nL of a solution consisting of 150 pg of Cas9 mRNA, 25 pg of sgRNA targeting intron 5 of *etv6*, 25 pg of universal sgRNA, and 10 pg of the donor plasmid into the cytosol of single-stage embryos of the *Tg(UAS:EGFP-CAAX)^m1230^ (UAS:GFP)* line [42]. The following day, we selected embryos displaying GFP fluorescence, nurtured them to maturity, and bred them with the *UAS:GFP* reporter line. Founder fish were identified by producing F1 offspring exhibiting green fluorescence in tissues known to express *etv6* mRNA [43]. The integration of the gene-breaking cassette was further confirmed in these offspring by first using PCR to amplify specific genomic regions and then subjecting the amplified DNA to Sanger sequencing (Figure S4 and Table S5). GFP-positive F1 offspring were raised to adulthood and used for further breeding with the *UAS:GFP* line to produce GPF-positive F2 families, which were continually monitored for leukemia development.

To establish the F0 generation *E::R;pax5*mut cohort harboring the *E::R* knock-in and a *pax5* mutation, we delivered two sgRNAs (25 pg each), targeting either exon 3 or exon 5 of *pax5*, along with 300 pg of Cas9 mRNA in a 2 nL volume to one-cell stage embryos derived from a cross between the *E::R* zebrafish line and the *UAS:GFP* line via microinjection. The following day, we verified successful mutagenesis in some of the injected embryos by using the T7 endonuclease I assay (Table S4) [41]. From the remaining embryos, we selected those that were GFP-positive, raised them to adulthood, and monitored them for the development of tumors.

To create the *E::R;cdkn2a/b+/−* zebrafish line, we initially generated a *cdkn2a/b+/−* zebrafish line. This was accomplished by microinjecting 2 nL of a solution containing three sgRNAs targeting the exon 2 of *cdkn2a/b* (each sgRNA at 12 pg) alongside 300 pg of Cas9 mRNA into single-cell-stage embryos from the *UAS:GFP* line (Table S2). F1 generation *cdkn2a/b+/−* zebrafish were established using standard procedures [44]. They carry a frameshift mutation in one allele of the *cdkn2a/b* gene, resulting in a premature stop codon after 210 nucleotides (Figure S5). These F1 fish were subsequently bred with the *E::R* zebrafish line to establish a stock of *E::R;cdkn2a/b+/−* zebrafish. The *E::R;cdkn2a/b+/−* fish line was inspected regularly for signs of leukemia.

### 2.4. Flow Cytometry Analysis

Entire kidneys were obtained from zebrafish and subsequently disintegrated through grinding between two etched glass slides. The material was then rinsed into a Petri dish with ice-cold 0.9× PBS containing 5% FBS (Gibco, Thermo Fisher Scientific, Waltham, MA, USA) (flow cytometry buffer). This suspension was subsequently passed through a 35 μm cell-strainer cap into a 5 mL tube to remove debris and obtain a solution of individual cells. From there, the single-cell suspensions were transferred into 1.5 mL Eppendorf tubes and pelleted by centrifugation (1200× *g*; 7 min; 4 °C). Cells were washed twice more in flow cytometry buffer and then stained with propidium iodide in flow cytometry buffer to facilitate the exclusion of dead cells (1 μg/mL). Next, cells were differentiated by light scatter features as outlined below (CytoFLEX, CytExpert v2.5, Brea, CA, USA): forward scatter (FSC)^low^ corresponds to mature erythroid cells; FSC^high^ and side scatter (SSC)^high^ correspond to myelomonocytes (consisting of neutrophils, monocytes, macrophages, and eosinophils); FSC^intermediate (int)^ and SSC^low^ contain lymphocyte cells (B lymphocytes, lymphoid progenitors, and hematopoietic stem cells). FSC^int^ and SSC^int^ contain immature progenitors (myeloid, lymphoid, and erythroid precursors). All quantifications were carried out using FlowJo software (v. 10.8.1, BD Biosciences, Franklin Lakes, NJ, USA) [45].

### 2.5. Giemsa Staining of Peripheral Blood Smears of Adult Zebrafish

After euthanizing the zebrafish using a 300 mg/L MS-222 (Sigma-Aldrich, Saint Louis, MO, USA) solution, a small incision was made in the caudal vein with a scalpel to collect a blood sample, which was anticoagulated using a 3.8% sodium citrate solution (Sigma-Aldrich, Saint Louis, MO, USA). Subsequently, a thin blood smear was prepared on a glass slide and allowed to air-dry for 30 min. Then, the smear was fixed in methanol (Sigma-Aldrich, Saint Louis, MO, USA) for 7 min and subsequently air-dried until the methanol had evaporated. For staining, a Giemsa (Sigma-Aldrich, Saint Louis, MO, USA) working solution was prepared by diluting the stock solution 1 to 9 in Milli-Q H_2_O and applying it to the smear, allowing it to incubate for 1 h. Following staining, the sample was observed under a Zeiss Axio Scope.A1 microscope (Zeiss, Oberkochen, Germany) and lymphoblasts were identified by their characteristically high nuclear-cytoplasmic ratio. Pictures were captured using an Axiocam 506 color camera (Zeiss, Oberkochen, Germany), and image acquisition was performed using ZEN 3.0 software (Zeiss, Oberkochen, Germany). Subsequently, images were processed with Fiji (ImageJ, v. 1.59i).

### 2.6. Hematoxylin and Eosin Staining on Paraffin Sections of Whole Adult Zebrafish

Zebrafish were euthanized as described in Section 2.5. and subsequently fixed in a 4% paraformaldehyde (Sigma-Aldrich, Saint Louis, MO, USA) solution overnight at 4 °C with continuous agitation. Following fixation, the fish were immersed in a 20% EDTA (Sigma-Aldrich, Saint Louis, MO, USA) solution (20 g of EDTA per 100 mL of PBS, pH adjusted to 7.2–7.3 with NaOH) for decalcification. This process lasted for 8 days at room temperature. In the following, the zebrafish were thoroughly rinsed with Milli-Q H_2_O and then dehydrated through a series of alcohol baths before being embedded in paraffin wax [46]. Subsequently, sagittal sections of the fish were cut to a thickness of 5 µm using a microtome.

To highlight cellular structures, we stained the sections with Hematoxylin and Eosin (Sigma-Aldrich, Saint Louis, MO, USA). The process involved immersing the sections in a Hematoxylin solution for approximately 5 min to selectively stain nuclei blue–purple, followed by counterstaining with Eosin for about 2 min to color the cytoplasm and other structures. After staining, the sections were dehydrated, cleared in xylene, and subsequently examined under a Zeiss Axio Scope.A1 microscope. Images of different tissues were captured using an Axiocam 506 color camera. Image acquisition and processing were performed as described in Section 2.5.

### 2.7. Western Blotting

Nuclear proteins from kidney marrow cells were extracted using the Thermo Scientific™ NE-PER Nuclear and Cytoplasmic Extraction Kit (Thermo Fisher Scientific, Waltham, MA, USA) following the instructions of the manufacturer. After extraction, these proteins were electrophoresed on Mini-PROTEAN^®^ TGX Stain-Free™ Precast 10% gels (Bio-Rad, Hercules, CA, USA), transferred onto a 0.2 µm PVDF membrane using the Trans-Blot Turbo Transfer System (Bio-Rad, Hercules, CA, USA), and processed using standard methods. As primary antibodies, rabbit anti-human RUNX1 antibody (1:400 in 5% BSA, ab 23980, Abcam, Cambridge, UK) and rabbit anti-β-Actin (1:60,000 in 5% BSA, sc-1615-R; Santa Cruz Biotechnology, Santa Cruz, CA, USA) were used. As a secondary antibody, the goat anti-rabbit IgG antibody was conjugated to Horseradish Peroxidase (1:7000 in 5% BSA, #7074S, Cell Signaling Technology, Danvers, MA, USA). Chemiluminescence was initiated with Cell Amersham ECL Reagent (GE Healthcare, Chicago, IL, USA) and detected using ChemiDocTM XRS+ with Image LabTM Software (version 6.0.1, Bio-Rad, Hercules, CA, USA). Protein sizing relied on the PageRuler Plus prestained ladder (Thermo Fisher Scientific, Waltham, MA, USA), and image processing was performed with Fiji (ImageJ, v. 1.59i).

### 2.8. Isolation of Total RNA from Whole Zebrafish and Kidney Tissue

We employed TRI Reagent (Sigma-Aldrich, St. Louis, MO, USA), following the manufacturer’s guidelines, to isolate total RNA from kidney tissue and whole zebrafish (30 days post-fertilization). The RNA obtained was subsequently purified using the RNeasy Mini kit (Qiagen, Hilden, Germany), following the manual provided with the kit. On-column DNA digestion was performed using the RNase-free DNase set from Qiagen (Qiagen, Hilden, Germany), as recommended by the manufacturers. The total RNA was used for either transcriptomics or as the starting material in the synthesis of cDNA with iScript (Bio-Rad, Hercules, CA, USA) according to the manufacturer’s instructions.

### 2.9. Statistics and Language Editing

Statistical analyses were conducted using GraphPad Prism 8.0.2 (GraphPad, La Jolla, CA, USA) with two-tailed Student’s *t*-tests. The statistical significance was represented as ‘ns’ for no statistical significance, * *p* < 0.05, ** *p* < 0.01, and *** *p* < 0.001.

Large Language Models (LLM) were used for English language editing.

### 2.10. Zebrafish Maintenance

Zebrafish were maintained and provided by the Tampere Zebrafish Core Facility (Tampere University, Tampere, Finland) at standard conditions [47,48].

### 2.11. RNA Sequencing and Bioinformatics Analysis

Library preparation and RNA sequencing of tumors were performed by Novogene (Novogene, Cambridge, UK). In addition, publicly available raw RNA-seq data for 103 samples from three separate zebrafish studies was downloaded from the Sequence Read Archive (SRA) by utilizing the sra-toolkit v. 2.11.1 to retrieve the fastq files [49,50,51].

Analysis of all RNA-seq data was performed by utilizing the nf-core/RNA-seq pipeline, version 3.12.0, which employs Nextflow v23.04.1 [52]. In summary, the analysis included quality control of read data with FastQC (v0.11.9), adapter, and quality trimming by Trim Galore! (v. 0.6.7) and read alignment to the reference genome GRCz10 by STAR (v. 2.6.1d), while quantification of gene expression was performed by utilizing Salmon. The merged gene counts generated by Salmon (v. 1.10.1) were further processed in R by utilizing the EdgeR package (v. 3.38.4), normalizing the data by using the trimmed mean of M-values (TMM) method, adjusting the data for library size, and finally performing log2 transformation for the counts per million (CPM)-scaled read counts. These steps were performed for the data from the present study (n = 11) as well as the data combined from the present study and the public datasets (n = 114). Genes lowly expressed in all cell types (B-ALL and healthy samples of the present study, and publicly available B-ALL, T-ALL, biphenotypic ALL, transplanted T-ALL, and thymocyte samples) were excluded by utilizing the FilterByExpr function.

Differential expression (DE) analyses were conducted by utilizing the limma R package (v3.52.4), determining the mean–variance relationship of the count data by using precision weights calculated by the voom function, while the lmFit function was used to fit a linear model to the expression value of each gene and the eBayes function to perform the empirical Bayes moderation on the linear model fit. DE analyses were performed for multiple different comparisons, including *E::R;pax5*mut leukemia samples versus healthy controls in the present study. We also compared the *E::R;pax5*mut zebrafish transcriptomes to the publicly available zebrafish T-ALL samples, as well as grouped them with the other zebrafish B-ALL samples to determine the DE genes between all (putative) zebrafish B-ALL and T-ALL transcriptomes. The significant DE genes were determined for each comparison by applying the adjusted *p*-value cutoff of 0.05. The DE genes were compared to B and T lineage genes collected from a variety of studies [49,50,51]. Both ComplexHeatmap (v. 2.12.1) and ggplot2 (v. 3.4.4) R packages were utilized in the visualization of gene expression data.

For comparison, the RNA-seq data of the 11 samples from the present study was also analyzed by utilizing the Galaxy platform, which enabled alignment to the zebrafish reference genome GRCz11 [53]. Similarly, the analysis included quality checks with FastQC, alignment of sequencing reads using STAR, quantification of gene expression with featureCounts, and DE analysis using DESeq2 [54,55]. Moreover, the GOseq Bioconductor package was utilized to determine enriched pathways and gene ontologies (GO) in the DE genes between the tumor and control samples [56].

## 3. Results

### 3.1. Generation of the ETV6::RUNX1 Zebrafish Model

**Figure 1:**
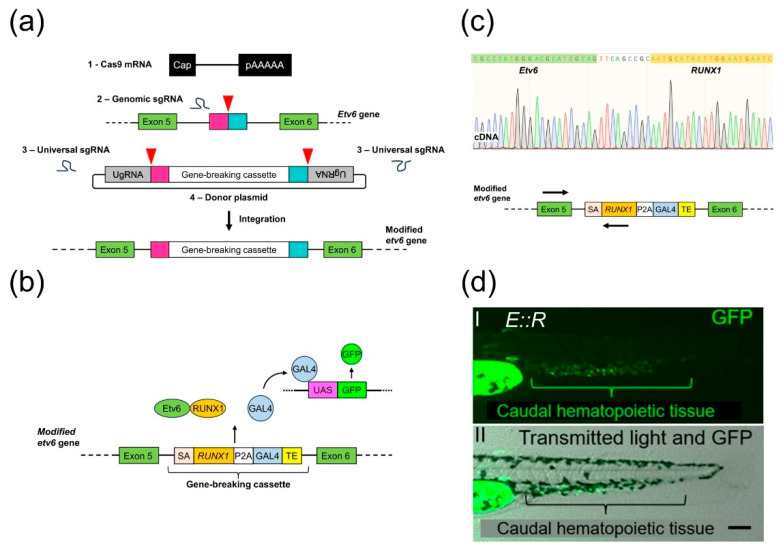
Generation of the *ETV6::RUNX1* (*E::R*) knock-in line. (**a**) A schematic illustrating the integration of the gene-breaking cassette into intron 5 of the *etv6* gene using the GeneWeld technique. The process required four reagents: (1) Cas9 mRNA, (2) genomic sgRNAs, (3) universal sgRNAs, and (4) a donor plasmid containing the gene-breaking cassette flanked by 48 bp homology arms and by universal sgRNA target sites. In vivo, Cas9-induced a targeted double-strand break (DSB) in the genome and generated two targeted DSBs in the donor plasmid, liberating the cassette along with the short homology arms. Subsequently, integration of the cassette followed. Red arrowheads: Cas9 nuclease-induced DSBs; pink and turquoise rectangles: homology arms; UgRNA: universal sgRNA target sites. (**b**) Schematic of the *E::R* zebrafish model showing the integrated gene-breaking cassette and the *UAS:GFP* transgene. *E::R* fusion protein and GAL4-VP16 protein expression are driven by the endogenous *etv6* promoter. GAL4-VP16 binds to UAS enhancer sequences, leading to GFP expression. SA: splice acceptor; TE: ocean pout antifreeze gene transcriptional termination and polyadenylation sequence. (**c**) Sequence of the in-frame splicing of the *etv6* and *RUNX1* transcripts. Primers used in the RT-PCR are either complementary to a region in exon 5 of *etv6* or the inserted human *RUNX1* cDNA, as indicated by arrows on the schematic of the *etv6* locus (Table S5). (**d**) Lateral view of a 2 day old *E::R* zebrafish larva with two images: (**d**(**I**)) GFP fluorescence in the caudal hematopoietic tissue and (**d**(**II**)) a merged image of GFP fluorescence with transmitted light, providing the structural context. Scale bar: 50 µm.

We aimed to generate a zebrafish line in which the expression of the *E::R* fusion gene is driven by the endogenous *etv6* promoter. We used the GeneWeld method, which combines the CRISPR/Cas9 system and a donor vector containing the transgene and short homology arms, to achieve precise integration of transgenes into the zebrafish genome (Figure 1a) [34,35]. Using this method, we introduced a gene-breaking cassette, SA-*RUNX1*-P2A-GAL4-TE, into intron 5 of the endogenous *etv6* gene. This cassette contains a segment of human *RUNX1* cDNA (exons 2–8) with a carp b-actin intron 1 splice acceptor positioned before it. As depicted in Figure 1b, downstream of the *RUNX1* cDNA, we inserted the coding sequence for a self-cleavable peptide, P2A, derived from porcine teschovirus-1, along with the cDNA of the transcriptional activator GAL4-VP16 and the transcriptional termination and polyadenylation sequence of the ocean pout antifreeze gene [57,58,59]. To generate *E::R* transgenic founders, the components of the GeneWeld method were introduced into one-cell stage embryos derived from the *UAS:GFP* line. A germline transmission rate of 1% (2 out of 208 fish) was achieved with precise integration of the 3440 bp long cassette in the F0 founders (Figure S4).

RT-PCR performed on cDNA derived from twenty whole 30 day old F1 generation *E::R* zebrafish confirmed the expression of the *E::R* fusion transcript. Sequencing further proved the faithful splicing of the *etv6* and *RUNX1* transcripts, resulting in an in-frame fusion transcript (Figure 1c). The GFP fluorescence pattern in F1 generation *E::R* embryos and larvae recapitulated the expression pattern of *etv6* mRNA recently observed in wild-type zebrafish by mRNA in situ hybridization [43]. GFP-positive cells were primarily located in tissues associated with primitive and definitive hematopoiesis, including the caudal hematopoietic tissue, which is analogous to the human fetal liver, the presumed site for the *t(12;21)(p13;q22)* translocation (Figure 1d and Figure S6) [60,61]. Hence, single-cell resolution analysis of GFP-positive cells suggested that expression of the *E::R* fusion was driven by the endogenous *etv6* promoter.

The GFP-expressing *E::R* zebrafish lines were propagated by crossing with the *UAS:GFP* line. Stable Mendelian transmission was observed for over three generations by monitoring the number of GFP-positive embryos in the clutches.

### 3.2. ETV6::RUNX1 Zebrafish Have an Expansion of the Precursor Cell Pool and a Low Incidence of Leukemia

**Figure 2:**
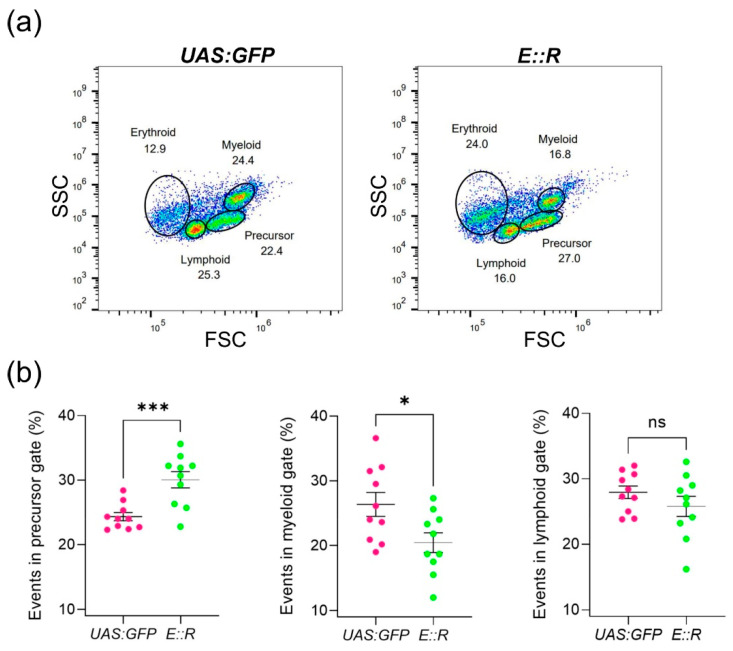
Flow cytometry analysis of major blood cell lineages in the whole kidney marrow of the *ETV6::RUNX1 (E::R)* zebrafish based on light scatter characteristics. (**a**) Flow cytometry plots representative of the *UAS:GFP* and *E::R* zebrafish lines are shown. (**b**) Scatter plots representing the percentage of cells in precursor, myeloid, and lymphoid fractions for the *E::R* (n = 10) and *UAS:GFP* zebrafish lines (n = 10). The statistical significance was represented as ‘ns’ for no statistical significance, * *p* < 0.05, and *** *p* < 0.001. All quantifications are presented as mean ± s.e.m.

Considering the substantial roles of the *ETV6* and *RUNX1* genes in blood cell development, we investigated whether the *E::R* zebrafish line exhibited any defects in hematopoietic differentiation [12,62]. To achieve this, we analyzed the whole kidney marrow of 4 to 5 month old fish from the *E::R* (F3 generation; n = 10) and *UAS:GFP* lines (n = 10) by using flow cytometry. To this end, we utilized light scatter properties that were recently assigned to each blood cell lineage [45]. Since erythrocytes were damaged during the preparation of single-cell suspensions, they were excluded before quantification with the FlowJo software (Figure S7). A statistically significant increase in the precursor cell population was observed in the kidney marrow of the *E::R* zebrafish compared to the *UAS:GFP* fish (*p* = 0.0008), while the myeloid cell fraction was diminished (*p* = 0.0238). No changes were observed in the lymphoid cell fraction (Figure 2).

Of the 102 *E::R* knock-in zebrafish, 2% (two fish) developed leukemia early, at 3 to 4 months of age, and none have developed leukemia since. This was confirmed by the presence of lymphoblasts in the peripheral blood smears (Figure S8). The low incidence of leukemia in our model mirrors the situation observed in newborns carrying the *E::R* fusion gene [6]. Notably, none of the several hundred *UAS:GFP* fish developed leukemia during the observation period.

### 3.3. CRISPR/Cas9-Induced Mutations in pax5 Increase Leukemia Incidence in the ETV6::RUNX1 Zebrafish Model

**Figure 3:**
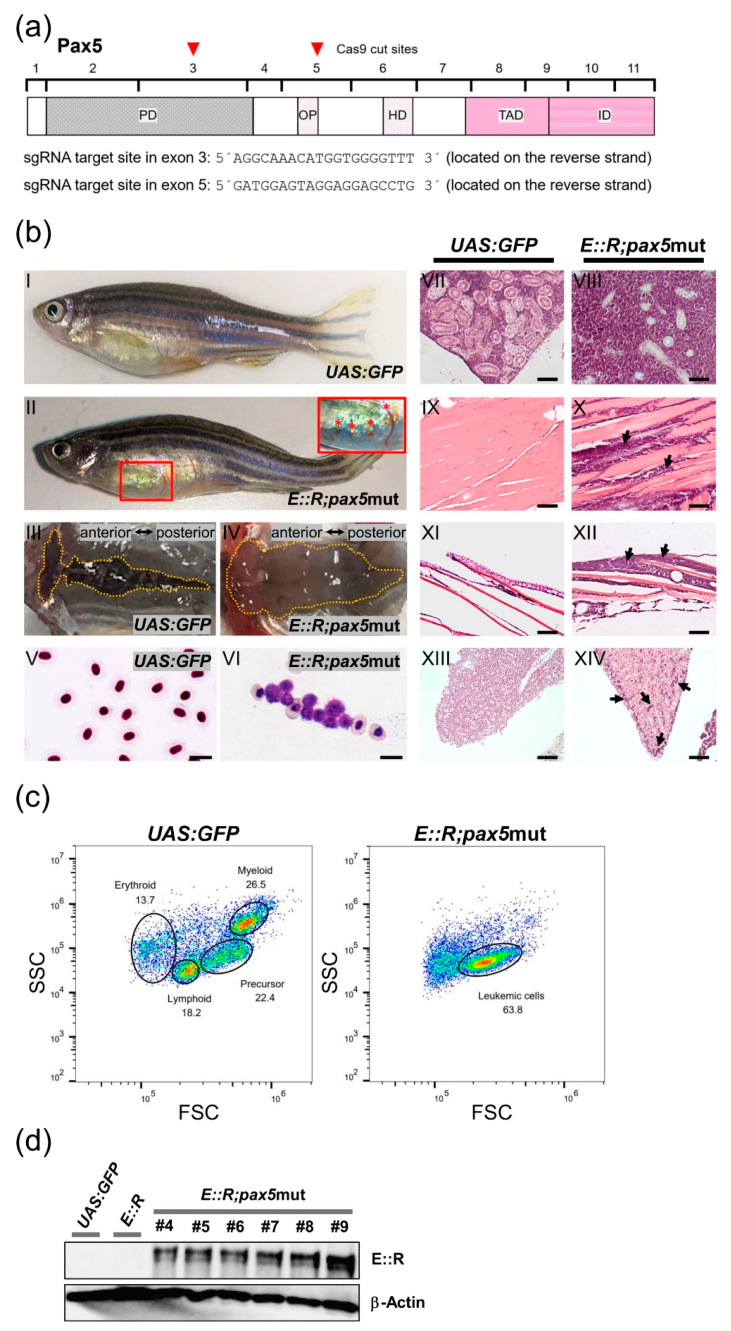
CRISPR/Cas9 targeting of the *pax5* gene and features of leukemic *E::R;pax5*mut fish. (**a**) Structure of the zebrafish Pax5 protein has five conserved functional domains: paired (PD), octapeptide (OP), homeo (HD), transactivation (TAD), and inhibitory (ID) domains. Brackets indicate the boundaries of the eleven encoding exons. Red arrowheads mark the Cas9 cut positions in exon 3 and exon 5, and the respective sequences of the sgRNA target sites are shown. (**b**) Phenotypic and histological analysis of *E::R;pax5*mut zebrafish. Images depict lateral views of representative control *UAS:GFP* zebrafish (**b**(**I**)) and leukemic zebrafish (**b**(**II**)). The *E::R;pax5*mut leukemic zebrafish developed subcutaneous bleedings in the ventral body region (highlighted by a red frame in **b**(**II**)). An inset provides a close-up view of the bleeding (marked with red asterisks) (**b**(**II**)). Top views of the entire kidneys of *UAS:GFP* and *E::R;pax5*mut leukemic zebrafish are shown (**b**(**III**–**IV**)). The kidney of the leukemic zebrafish exhibited enlargement along its entire length (**b**(**IV**)). Giemsa staining of a peripheral blood smear from the *UAS:GFP* fish (**b**(**V**)) revealed normal nucleated erythrocytes, while Giemsa staining of the leukemic blood smear (**b(VI**)) showed the presence of clusters of lymphoblasts. Hematoxylin and Eosin staining of the sagittal paraffin sections of tissues from *UAS:GFP* fish (**b**(**VII**,**IX**,**XI**,**XIII**)) and leukemic fish (**b**(**VIII**,**X**,**XII**,**XIV**)) highlighted the presence of lymphoblasts in the kidney marrow (**b**(**VIII**)), muscle tissue (**b**(**X**), arrows), epidermis (**b**(**XII**), arrows), and liver (**b**(**XIV**), arrows). Scale bars: (**b**(**V**,**VI**)): 10 µm; (**b**(**VII**–**XIV**)): 100 µm. (**c**) Flow cytometry plot (**right**) showing cell populations separated by their light scatter characteristics in the whole kidney marrow of leukemic *E::R;pax5*mut fish, along with the *UAS:GFP* control plot (**left**). The predominant cell population in the leukemic fish exhibited light scatter characteristics similar to the precursor cell fraction in the control group. (**d**) Western blot analysis was performed using protein extracted from kidney marrow cells of *UAS:GFP* fish, non-leukemic *E::R* knock-in fish, and 6 out of 10 leukemic *E::R;pax5*mut fish (#4–#9). Primary antibodies targeting human RUNX1 and β-Actin were used. E::R protein was detected in all tumor samples but not in the samples from the *UAS:GFP* control or non-leukemic *E::R* knock-in fish. The uncropped blots are shown in Supplementary Materials.

Given that additional genetic aberrations are required for the onset of *E::R* leukemia, we investigated whether deliberately introducing them affected the incidence of the disease [63,64]. The *PAX5* gene is deleted in about 25% of *E::R* patients and is one of the most commonly mutated genes in this subtype [15,16]. To mutate the zebrafish ortholog of *PAX5* in the *E::R* knock-in line, we injected two sgRNAs targeting either exon 3 or exon 5, which encode functional domains of the protein, and Cas9 mRNA into one-cell-stage embryos (Figure 3a).

In the F0 generation *E::R;pax5*mut cohort (n = 68), fifteen percent (n = 10) of fish developed an overt disease over a latency period of 9 to 16 months. Two fish were found deceased, while the remaining eight exhibited subcutaneous bleeding, altered swimming behavior, or were lying at the bottom of the tank (Figure 3b(II)).

To further characterize these zebrafish, we analyzed the kidney, the primary site of B-cell hematopoiesis [65,66]. A top-view examination revealed a pale and enlarged kidney compared to the kidney from the *UAS:GFP* fish (Figure 3b(III,IV)). Flow cytometry analysis of the kidney marrow revealed a large cell population exhibiting light scatter characteristics similar to precursor cells in the control *UAS:GFP* fish, indicating accumulation of precursor cells and impaired differentiation (Figure 3c). Subsequent histological analysis revealed that lymphoblast accumulation around the kidney tubules destroyed kidney architecture, causing kidney enlargement (Figure 3b(VIII)). Lymphoblasts were also found in non-hematopoietic tissues, such as muscle, liver, and epidermis (Figure 3b(X,XII,XIV)). Overt leukemia also manifests as clusters of lymphoblasts in the peripheral blood, as determined by Giemsa staining (Figure 3b(VI)). These characteristics suggest that the *E::R;pax5*mut zebrafish developed ALL that originated in the kidney marrow and subsequently spread to distant organs.

Western blot analysis using an antibody targeting the human RUNX1 protein confirmed that the leukemias were positive for the E::R fusion protein. Interestingly, the E::R fusion protein was undetectable in non-leukemic *E::R* knock-in fish (Figure 3d and Figure S9), suggesting that the fusion protein was weakly expressed. We used the Integrative Genomics Viewer (IGV) to analyze the mapped reads from the leukemia transcriptomes, confirming that the leukemic fish had deleterious mutations in exon 3 and exon 5 of the *pax5* gene (Figure S10).

### 3.4. A Frameshift Mutation in cdkn2a/b Increases Leukemia Incidence in the ETV6::RUNX1 Zebrafish Model

**Figure 4:**
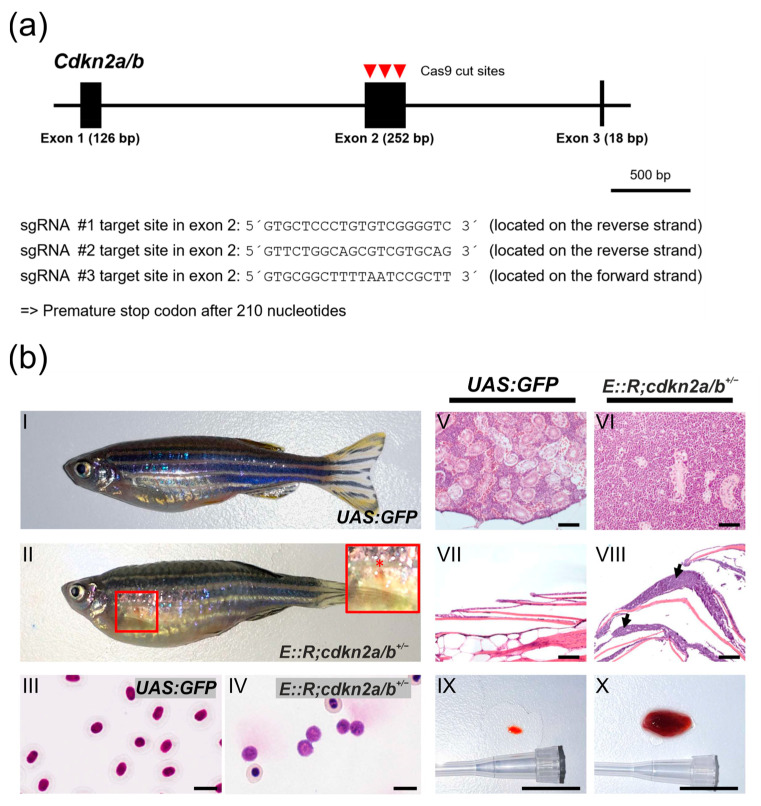
Generation of *cdkn2a/b^+/−^* zebrafish using the CRISPR/Cas9 genome editing and features of leukemic *E::R;cdkn2a/b^+/−^* fish. (**a**) Genomic structure of the *cdkn2a/b* gene. Red arrowheads mark the Cas9 cut positions in exon 2, and the sequences of the three sgRNA target sites are shown. The *cdkn2a/b^+/−^* zebrafish line with a premature stop codon after 210 nucleotides was established. (**b**) Phenotypic and histological characteristics of leukemic *E::R;cdkn2a/b^+/−^* zebrafish. Images depict lateral views of representative control *UAS:GFP* zebrafish (**b**(**I**)) and leukemic zebrafish (**b**(**II**)). The *E::R;cdkn2a/b^+/−^* leukemic zebrafish developed subcutaneous bleeding in the ventral body region (highlighted by a red frame in (**b**(**II**))). An inset provides a close-up view of the bleeding (marked with a red asterisk) (**b**(**II**)). Giemsa staining of a peripheral blood smear from the *UAS:GFP* fish (**b**(**III**)) revealed normal nucleated erythrocytes, while Giemsa staining of a leukemic blood smear (**b**(**IV**)) showed a significant presence of lymphoblasts. Hematoxylin and Eosin staining of sagittal paraffin sections of tissues from the *UAS:GFP* fish (**b**(**V**,**VII**)) and leukemic fish (**b**(**VI**,**VIII**)) revealed the presence of large deposits of lymphoblasts in the kidney marrow (**b**(**VI**)) and epidermis (**b**(**VIII**), arrows) of leukemic fish. The spleen was enlarged in the leukemic *E::R;cdkn2a/b^+/−^* fish (**b**(**X**)) compared to the *UAS:GFP* control fish (**b**(**IX**)). Scale bars: (**b**(**III**,**IV**)): 10 µm; (**b**(**V**–**VIII**)): 100 µm; (**b**(**IX**,**X**)): 1 cm.

Next, to investigate whether other frequently occurring lesions in *E::R*-positive childhood pB-ALL similarly increased the leukemia incidence, we mutated the zebrafish ortholog of the human *CDKN2A/B* gene in the *E::R* knock-in zebrafish line, as the *CDKN2A/B* gene is also affected by deletions in 25% of children with *E::R*-positive leukemia [15,16]. To achieve this, we used the CRISPR/Cas9 genome editing system to generate a *cdkn2a/b^+/−^* zebrafish line in the *UAS:GFP* background. In this *cdkn2a/b^+/−^* line, one of the *cdkn2a/b* alleles carries a premature stop codon, supposedly resulting in a loss of protein expression (Figure 4a) [67]. Subsequently, we crossed the *cdkn2a/b^+/−^* line with the *E::R* knock-in fish to establish the *E::R;cdkn2a/b^+/−^* double mutant fish line. At 9 to 11 months old, 7% (3/41) of the fish had small subcutaneous bleedings and were lying at the bottom of the tank (Figure 4b(II)). Histological analysis of an enlarged kidney revealed large deposits of lymphoblastic cells that destroyed the architecture of kidney tissue (Figure 4b(VI)). Tumor cell deposits were also found in distant tissues, such as the epidermis (Figure 4b(VIII)). Giemsa staining of peripheral blood smears revealed a significant number of lymphoblasts in the blood (Figure 4b(IV)), and the spleen was massively enlarged (Figure 4b(X)).

### 3.5. Transcriptomic Analyses Reveal That E::R;pax5mut Zebrafish Develop B-Lineage ALL

To investigate whether the *E::R;pax5*mut leukemias belonged to either B- or T-lymphoblastic lineages, we conducted transcriptomic analysis on eight samples and compared them to three transcriptomes derived from the kidney marrow of the non-leukemic *UAS:GFP* zebrafish (control), as well as to various transcriptomes available from previous zebrafish leukemia studies [49,50,51].

We identified significantly (adjusted *p*-value ≤ 0.05) differentially expressed (DE) genes between the control and *E::R;pax5*mut zebrafish and evaluated their association with cell lineage (Figure S11 and Table S6). Consistent with high B-lineage leukemia infiltration, many B-cell-associated genes such as *ebf1*, *cd79a*, *cd79b*, and *syk* were expressed in the *E::R;pax5*mut zebrafish at significantly higher levels compared to the control fish kidney tissue. *Pax5* transcripts with the targeted mutations were predominant (Figure S11 and Table S6). The expression of the immature lymphocyte-associated gene *rag2*, in turn, was significantly higher in the leukemic *E::R;pax5*mut zebrafish. In line with this, *rag1* expression was also on average high in the leukemic samples; however, it was more variable (at the control level in tumors #6 and #8) (Figure S12). The proximity of the transcriptome of tumor #8 that resembled the controls may indicate a lower leukemic cell fraction.

**Figure 5:**
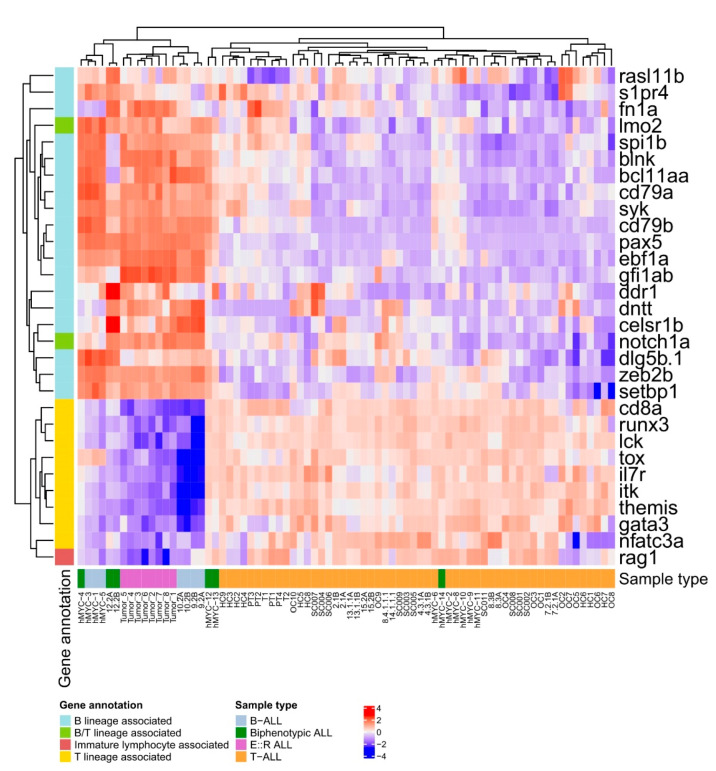
Heatmap visualization of the significantly differentially expressed B and T lineage-associated genes between the B-ALL and *E::R;pax5*mut versus T-ALL zebrafish transcriptomes and their expression across the different zebrafish leukemia types (adjusted *p*-value ≤ 0.05). B-ALL and *E::R;pax5*mut zebrafish leukemias expressed several B-lineage-associated genes at significantly higher levels compared to zebrafish with T-ALL, while the expression difference was the opposite for multiple T lineage-associated genes.

To further assess whether the *E::R;pax5*mut zebrafish leukemias better resembled B- or T-lymphoblastic leukemias, we downloaded publicly available transcriptomic data from the *rag2-hMYC*-driven and *rag2-mMyc*-driven zebrafish leukemia models, both yielding a mixture of B-ALL, biphenotypic ALL and T-ALL leukemias, as well as from a previous *rag2-TLX1*-driven T-ALL zebrafish model [49,50,51]. DE analyses comparing the current *E::R;pax5*mut and the publicly available zebrafish B-ALL samples against the T-ALL zebrafish models revealed that the *E::R;pax5*mut samples clustered closely together with B-lineage and biphenotypic ALLs, expressing B-lineage-associated genes, including *pax5*, *ebf1a*, *cd79a*, *cd79b*, and *syk*, at significantly higher levels compared to the T-ALL fish. In contrast, the T cell-associated genes, like *runx3*, *il7r*, *nfatc3a*, and *tox*, had significantly higher expression in the zebrafish T-ALLs (Figure 5, Figure S13, and Table S7). Interestingly, *rag1*, which is associated with immature lymphocytes, had higher expression in T-ALLs, while *notch1a* and *lmo2*, which are associated with both B and T lineages, were expressed at higher levels among the B-ALL and *E::R;pax5*mut zebrafish (Figure 5, Figure S13, and Table S7). When these transcriptomic signatures were compared between our *E::R;pax5*mut and the T-ALL fish, a very similar expression pattern was obtained, i.e., levels of B-lineage-associated genes were higher and T cell-associated genes were lower in the *E::R;pax5*mut fish (Figure S14 and Table S8). Taken together, transcriptomic signatures suggest that the generated *E::R;pax5*mut zebrafish represent B-lineage ALL and are readily distinguishable from the T-ALLs.

In *E::R;pax5*mut leukemias, enrichment of the KEGG pathways was determined through gene set enrichment analysis using Goseq [56]. We observed a significant enrichment of pathways related to DNA maintenance and repair and metabolic regulation (Table S9), both intimately involved in the pathogenesis of leukemia.

## 4. Discussion

The *E::R* subtype is the second most common form of pB-ALL in children after high hyperdiploidy. With contemporary chemotherapy, the outcome is usually favorable, yet a significant subset of patients experience disease recurrence. Animal models that faithfully recapitulate human disease are needed to fully understand the resistance mechanisms. Hence, we generated a zebrafish model of *E::R* leukemia by using CRISPR-Cas9 genome editing technology and introduced targeted lesions in two frequently mutated genes in this leukemia subtype. Our results show that leukemia incidence is significantly increased by secondary lesions in the *pax5* and *cdkn2a/b* genes. The transcriptomic analysis confirmed that the *E::R;pax5*mut leukemias represented exclusively the B-lineage ALL. Our novel animal model provides a unique opportunity to further elucidate the disease pathogenesis and genetic features that impact drug sensitivity.

Therapy response, as measured by minimal residual disease, is the most significant prognostic factor in ALL [68]. A subset of *E::R* leukemias have inadequate early therapy responses, subsequently increasing the risk of relapse [68]. The reasons behind the treatment resistance are unknown, but the genomic heterogeneity of the disease is one of the prime candidates. The literature is conflicting regarding the contribution of secondary mutations to the risk of relapse in genes such as *CDKN2A/B* or *ETV6* [69,70]. Interpretation of such data is markedly complicated by the heterogeneous mixture of mutations at disease presentation. The same problem exists for cell culture models, such as REH cells, and patient-derived xenografts.

Zebrafish offer a valuable platform for developing a wide range of leukemia models, thanks to their adaptable genetics through CRISPR/Cas9, high fecundity, and conserved hematopoietic processes. Sabaawy et al. pioneered a zebrafish model for childhood *E::R* pB-ALL [31]. They established two transgenic zebrafish lines by randomly integrating transgenes, wherein the expression of the human E::R fusion gene was driven by either the Xenopus elongation factor 1α or the zebrafish β-actin promoter [31]. A significant limitation was the ubiquitous expression of the E::R fusion protein, deviating from natural locations and levels. This weakened the ability of their model to replicate human disease and led to unintended consequences, such as the development of fatal lymphoid hyperplasia observed in 6% of the zebrafish [31].

To address previous shortcomings, we developed a new model in which the *E::R* fusion gene was expressed under the control of the endogenous *etv6* promoter. This was performed by inserting the human *RUNX1* cDNA-containing gene-breaking cassette into intron 5 of the zebrafish *etv6* gene using the GeneWeld method [34,35]. This method has previously been used in zebrafish to generate fluorescent reporter lines, Cre recombinase driver lines, and Cre/lox-responsive conditional alleles [34,71,72]. We achieved a lower success rate of 1%, but similar gene-breaking cassettes have previously been used effectively in applications such as gene-breaking transposon mutagenesis in zebrafish [37,58,73,74]. Liu and colleagues reported that by incorporating gene-breaking cassettes into the introns of genes, they were able to disrupt gene expression with an over 99% reduction in normal transcript levels [72]. Epigenetic mechanisms like altered histone modifications and DNA methylation patterns drive enhancer hijacking—a key feature in certain cancers [75,76]. Our approach, combining the GeneWeld technique and elements of gene-breaking cassettes, could be used to create zebrafish models of cancer that mimic this process by placing oncogenes under the control of respective enhancers, offering a concise method to replicate the regulatory alterations associated with enhancer hijacking and gain insights into cancer mechanisms.

The *E::R* fusion gene was detected in non-leukemic *E::R* knock-in fish by RT-PCR, but the fusion protein was only detected in leukemic fish. This is similar to the challenges of detecting the E::R fusion protein in human newborns [77,78]. Our results show that the fusion protein is expressed only in *etv6-expressing* cells and only at low physiological levels. This is a strength of our model, and it may also explain why none of our zebrafish developed fatal lymphoid hyperplasia, as described in the previous model [31]. Our *E::R* knock-in zebrafish model has another advantage over the previous model: it enables the detection of fusion protein expression through single-cell resolution GFP fluorescence. Because zebrafish develop outside the womb (ex utero), our model is the first to enable the observation of *E::R*-positive cell dynamics in the caudal hematopoietic tissue, which is analogous to the human fetal liver, the presumed site where the translocation occurs in the body [60,61].

Analysis of the major blood lineages in the kidney marrow, the counterpart to human bone marrow, of *E::R* knock-in zebrafish revealed a significant increase in the precursor cell population. This finding aligns with the observations of Sabaawy et al., who linked the expansion of the precursor cell pool to disruption in B-cell maturation [31]. Likewise, in the *Sca1*-driven mouse model for *E::R*, Rodríguez-Hernández et al. observed an aberrant B-cell compartment [79]. This is also the case in humans, where similar expansion of the precursor cell pool has been noted and presumed to precede the occurrence of overt disease [80].

Two percent of the *E::R* knock-in zebrafish developed leukemia, which is consistent with the frequency observed in human carriers of the fusion gene [6]. This low rate suggests that the E::R fusion protein is a weak oncogene and that additional mutations are necessary for leukemogenesis [63,64]. Indeed, when additional mutations were separately introduced into the zebrafish orthologs of the human *PAX5* and *CDKN2A/B* genes, which are deleted in a quarter of the *E::R* leukemias, the incidence increased to 15% and 7%, respectively, which is similar to what has been observed in mice [14,15,16,81]. Transcriptomics analyses revealed that the *E::R* knock-in zebrafish mutated in the *pax5* gene developed B-lineage ALL, as indicated by high expression of the B-cell genes *cd79b*, *pax5*, *blnk*, and *ebf1*. These genes were also upregulated in B-lineage ALL from the *rag2:mMyc* and *rag2:hMYC;lck:eGFP* zebrafish models [50]. Differentially expressed genes also included *rag1* and *rag2*, which are hallmarks of human *E::R*-positive leukemia (MILE study; http://r2.amc.nl; accessed on the 7 September 2023) [82]. Overall, these results highlight the similarities of our animal model to human *E::R*-positive leukemia and contrast it with respective mouse models, which often produce a mixture of leukemias from different cell lineages [12,13,83].

In discussing our findings, it is crucial to acknowledge certain limitations. Our study of *E::R;pax5*mut and *E::R;cdkn2a/b* leukemias lacks control data without the *E::R* fusion gene. Additionally, a detailed analysis of the genomic features of the two *E::R*-positive leukemias that did not harbor targeted secondary lesions was not available.

## 5. Conclusions

In conclusion, we have developed a zebrafish model for *E::R* leukemia that recapitulates many features of human disease. This model provides a valuable platform for investigating disease pathogenesis, the individual contributions of secondary mutations, and their relationship to drug sensitivity. It has the potential to inform personalized treatment strategies for childhood B-ALL.

## Data Availability

The datasets generated and analyzed during the current study are available in the Sequence Read Archive (BioProject accession number: PRJNA1038711, https://www.ncbi.nlm.nih.gov/bioproject/1038711; registered on the 10 November 2023). Publicly available raw RNA-seq data from zebrafish ALLs analyzed in the current study were downloaded from the Sequence Read Archive (BioProject accession numbers: PRJNA637328 and PRJNA488354, https://www.ncbi.nlm.nih.gov/bioproject/PRJNA637328, https://www.ncbi.nlm.nih.gov/bioproject/PRJNA488354; both BioProjects were accessed on the 12 October 2023) [49,50].

## Supplementary Materials

The following supporting information can be downloaded at: https://www.mdpi.com/article/10.3390/cancers15245821/s1, Method S1: Detailed description of the generation of the donor plasmid, Method S2: Identification of intron 5 sequences of zebrafish *etv6* in the *UAS:GFP* line, Figure S1: Sequence of the donor plasmid, Figure S2: Sequences of intron 5 of *etv6*, Figure S3: Homology arm design, Figure S4: Chromatogram of Sanger sequencing of the genomic integration, Figure S5: Sanger sequencing analysis of exon 2 of the *cdkn2a/b* gene in the *E::R;cdkn2a/b^+/−^* zebrafish line, Figure S6: GFP Expression in the *E::R* knock-in line during primitive hematopoiesis, Figure S7: Flow cytometry plots, Figure S8: Giemsa staining of peripheral blood shows a high nuclear–cytoplasmic ratio of the lymphoblasts in the *E::R* knock-in fish, Figure S9: Uncropped Western blot of *UAS:GFP*, non-leukemic *E::R* and leukemic *E::R;pax5*mut zebrafish lines by using the human anti-RUNX1 antibody, Figure S10: Integrative Genomics Viewer screenshots illustrating mutations in *pax5* exon 3 and exon 5 in *E::R;pax5*mut zebrafish, Figure S11: Heatmap visualizing the significantly differentially expressed (adjusted *p*-value ≤ 0.05) B and T lineage-associated genes between the leukemic *E::R;pax5*mut and non-leukemic kidney marrow zebrafish transcriptomes, and their expression across the two groups, Figure S12: Heatmap visualizing the expression of *rag1* and *rag2* genes across the leukemic *E::R;pax5*mut and non-leukemic kidney marrow zebrafish transcriptomes, Figure S13: Boxplots describing the gene expression level of B and T lineage-associated genes, Figure S14: Heatmap visualizing the significantly differentially expressed (adjusted *p*-value ≤ 0.05) B and T lineage-associated genes between the *E::R;pax5*mut ALL and T-ALL zebrafish transcriptomes and their expression across the two different zebrafish leukemia types, Table S1: Oligos and templates used for the construction of the donor plasmid, Table S2: Targets of sgRNAs, Table S3: Oligos for the generation of sgRNA templates, Table S4: Oligos used for T7 endonuclease I assays, Table S5: Oligos used to amplify the genomic region over the insertion site of the gene-breaking cassette or a piece of the *E::R* cDNA, Table S6: Significantly differentially expressed B and T lineage-associated genes between the leukemic *E::R;pax5*mut and non-leukemic kidney marrow zebrafish transcriptomes and their adjusted *p*-values, Table S7: Significantly differentially expressed B and T lineage-associated genes between *E::R* ALL, B-ALL, biphenotypic ALL, and T-ALL zebrafish transcriptomes and their adjusted *p*-values, Table S8: Significantly differentially expressed B and T lineage-associated genes between *E::R* ALL and T-ALL zebrafish transcriptomes and their adjusted *p*-values, Table S9: Enriched KEGG pathways in *E::R;pax5*mut tumors, Table S10: Oligos used to amplify pieces of the *etv6* intron 5 via PCR.
