# Supplementary material for: Generation of a Zebrafish Knock-In Model Recapitulating Childhood ETV6::RUNX1-Positive B-Cell Precursor Acute Lymphoblastic Leukemia
Source: Cancers (Basel). 2023 Dec 13;15(24):5821. doi: 10.3390/cancers15245821 (PMC10871125; doi:10.3390/cancers15245821)
Supplement: Supplementary file 1 [file cancers-15-05821-s001.zip › cancers-2756528-supplementary.pdf]

Supplementary Materials

Method S1. Detailed description of the generation of the donor plasmid

The *pGTag-TagRFP-B-actin* vector (Addgene #117808) was opened using the *XbaI* and *EcoRI* restriction enzymes. Next, annealed oligos were integrated to remove the *XbaI* restriction site, which could have hindered subsequent vector assembly. Subsequently, the insert *TagRFP-B-actin-terminator* was excised from the vector through the utilization of *BamHI* and *NcoI* restriction enzymes. Annealed oligos (*ClaI-NotI-SacII-SpeI*-P2A-*XbaI-NcoI*) containing essential restriction sites for future vector assembly together with the P2A sequence were ligated into the opened vector. Following this, the vector was cleaved with *XbaI* and *NcoI*, and two PCR fragments harboring either the GAL4-VP16 cDNA or the ocean pout antifreeze gene transcriptional termination and polyadenylation sequence were simultaneously incorporated. The templates for the generation of these PCR fragments were the vectors *PME-Gal4VP16* and *pGBT-RP2-1* (Addgene #31828), respectively [1,2]. Subsequently, a PCR fragment of human *RUNX1* cDNA spanning from exon 2 to exon 8 was ligated into the vector utilizing the *SacII* and *SpeI* restriction enzymes. As a template for the PCR served a plasmid containing the full *RUNX1* cDNA (Origene; #SC123977). Lastly, the carp b-actin intron 1 splice acceptor was amplified by PCR using the *pGBT-RP2-1* plasmid as a template and ligated into the plasmid by using the *SacII* and *XbaI* restriction enzyme sites (*XbaI* site is present in the partial *RUNX1* cDNA) (Table S1 and Figure S1).

Method S2. Identification of intron 5 sequences of zebrafish etv6 in the UAS:GFP line

The intron 5 sequences of zebrafish *etv6* in the *UAS:GFP* line, which were crucial for donor plasmid assembly, were identified based on a short segment of intron 5 of the *etv6* gene, which is present in a genomic scaffold within the Zv8 zebrafish assembly (Zv8_NA6723:-5971:16082:-1). This region was amplified by using primers that are directly adjacent to exon 5 in the *etv6* alleles of the *UAS:GFP* line (Table S10). After amplification, we subcloned the pieces into the vector *pME-MCS* and Sanger sequenced the fragments [1]. Sequence 1 was used as the reference for creating both sgRNA and homologous arms (Figure S2).

References

1. Kwan, K.M.; Fujimoto, E.; Grabher, C.; Mangum, B.D.; Hardy, M.E.; Campbell, D.S.; Parant, J.M.; Yost, H.J.; Kanki, J.P.; Chien, C.-B. The Tol2kit: A Multisite Gateway-Based Construction Kit for Tol2 Transposon Transgenesis Constructs. *Dev Dyn* **2007**, *236*, 3088–3099, doi:10.1002/dvdy.21343.
2. Clark, K.J.; Balciunas, D.; Pogoda, H.-M.; Ding, Y.; Westcot, S.E.; Bedell, V.M.; Greenwood, T.M.; Urban, M.D.; Skuster, K.J.; Petzold, A.M.; et al. In Vivo Protein Trapping Produces a Functional Expression Codex of the Vertebrate Proteome. *Nat Methods* **2011**, *8*, 506–515, doi:10.1038/nmeth.1606.

UgRNA target site:

GGGAGGCGTTCGGGCCACAGCGG

PAM

***BfuAI* cut site: ACCTG(4/8)**

***BsQpI* cut site: GCTCTTC(1/4)**

Carp β-actin intron 1 splice acceptor: NNNNNN

Human *RUNX1* cDNA exon 2-8: NNNNNN

P2A coding sequence: NNNNNN

GAL4-VP16 cDNA: NNNNNN

Ocean pout antifreeze poly(A) signal; terminator; putative border element: NNNNNN

```
CTTTCCTGCGTTATCCCCTGATTCTGTGGATAACCGTATTACCGCCTTTGAGTGAGCTGATACCGCTCGCCGCAGCCGAACGACCGAGCGCAGCGAGTCAGTGAGCGAGGAAGCGGATGTGCGCCCA
ATACGCAAACCGCCTCTCCCCGCGCGTTGGCCGATTCATTAATGCAGCTGGCACGACAGGTTTCCCGACTGGAAAGCGGGCAGTGAGCGCAACGCAATTAATACGCGTACCGCTAGCCAGGAAGAGTTTGTAG
AAACGCAAAAAGGCCATCCGTCAGGATGGCCTTCTGCTTAGTTTGATGCCTGGCAGTTTATGGCGGGCGTCCTGCCCGCCACCCTCCGGGCCGTTGCTTCACAACGTTCAAATCCGCTCCCGGCGGATTTGTC
CTACTCAGGAGAGCGTTCACCGACAAACAACAGATAAAACGAAAGGCCCAGTCTTCCGACTGAGCCTTTCGTTTTATTTGATGCCTGGCAGTTCCCTACTCTCGCGTTAACGCTAGCATGGATGTTTTCCCAG
TCACGACGTTGTAAAACGACGGCCAGTCTTAAGCTCGGGCCCTGCAGCTCTAGTAGGGATAACAGGGTAATAAATTCGGGAGGCGTTCGGGCCACAGCGGACACGCAGGTGCGCAACGCAATTAATGTGAGTT
AGCTCACTCATTAGGCACCCCAGGCTTTACACTTTATGCTTCCGGCTCGTATGTTGTGTGGAATTGTGAGCGGATAACAATTTCACACAGGAAACAGCTATGACCATGATTACGCCAAGCGCGCAATTAACCC
TCACTAAAGGGAACAAAAGCTGGAGGACCTCGAGGGGGGGCCCGGTACCCAATTCGCCCTATAGTGAGTCGTATTACGCGCGCTCACTGGCCGTCGTTTTACAACGTCGTGACTGGGAAAACCCTGGCGTTAC
CCAACTTAATCGCCTTGCAGCACATCCCCCTTTCGCCAGCTGGCGTAATAGCGAAGAGGCCCGCACCGATCGCCCTTCCCAACAGTTGCGCAGCCTGAATGGCGAATGGACCTGCCACAGGATCTATCGATGC
GGCCGCCCGCGGCACCCACTAGCGGTCAGACTGCAGATTGCAGCACGAAACAGGAAGCTGACTCCACATGGTCACATGCTCACTGAAGTGTTGACTTCCCTGACAGCTGTGCACTTTCTAAACCGGTTTTCTC
ATTCATTTACAGTTCAGCCGCAATGCATACTTGGAATGAATCCTTCTAGAGACGTCCACGATGCCAGCACGAGCCGCCGCTTCACGCCGCCTTCCACCGCGCTGAGCCCAGGCAAGATGAGCGAGGCGTTGCC
GCTGGGCGCCCCGGACGCCGGCGCTGCCCTGGCCGGCAAGCTGAGGAGCGGCGACCGCAGCATGGTGGAGGTGCTGGCCGACCACCCGGGCGAGCTGGTGCGCACCGACAGCCCCAACTTCCTCTGCTCCGTG
CTGCCTACGCACTGGCGCTGCAACAAGACCCTGCCCATCGCTTTCAAGGTGGTGGCCCTAGGGGATGTTCCAGATGGCACTCTGGTCACTGTGATGGCTGGCAATGATGAAAACTACTCGGCTGAGCTGAGAA
ATGCTACCGCAGCCATGAAGAACCAGGTTGCAAGATTTAATGACCTCAGGTTTGTCGGTCGAAGTGGAAGAGGGAAAAGCTTCACTCTGACCATCACTGTCTTCACAAACCCACCGCAAGTCGCCACCTACCA
CAGAGCCATCAAAATCACAGTGGATGGGCCCCGAGAACCTCGAAGACATCGGCAGAAACTAGATGATCAGACCAAGCCCGGGAGCTTGTCCTTTTCCGAGCGGCTCAGTGAACTGGAGCAGCTGCGGCGCACA
GCCATGAGGGTCAGCCCACACCACCCAGCCCCACGCCCAACCCTCGTGCCTCCCTGAACCACTCCACTGCCTTTAACCCTCAGCCTCAGAGTCAGATGCAGGATACAAGGCAGATCCAACCATCCCCACCGT
GGTCCTACGATCAGTCCTACCAATACCTGGGATCCATTGCCTCTCCTTCTGTGCACCCAGCAACGCCCATTTCACCTGGACGTGCCAGCGGCATGACAACCCTCTCTGCAGAACTTTCCAGTCGACTCTCAAC
GGCACCCGACCTGACAGCGTTCAGCGACCCGCGCCAGTTCCCCGCGCTGCCCTCCATCTCCGACCCCCGCATGCACTATCCAGGCGCCTTCACCTACTCCCCGACGCCGGTCACCTCGGGCATCGGCATCGGC
ATGTCGGCCATGGGCTCGGCCACGCGCTACCACACCTACCTGCCGCCGCCCTACCCCGGCTCGTCGCAAGCGCAGGGAGGCCCGTTCCAAGCCAGCTCGCCCTCCTACCACCTGTACTACGGCGCCTCGGCCG
GCTCCTACCAGTTCTCCATGGTGGGCGGCGAGCGCTCGCCGCCGCGCATCCTGCCGCCCTGCACCAACGCCTCCACCGGCTCCGCGCTGCTCAACCCCAGCCTCCCGAACCAGAGCGACGTGGTGGAGGCCGA
GGGCAGCCACAGCAACTCCCCCACCAACATGGCGCCCTCCGCGCGCCTGGAGGAGGCCGTGTGGAGGCCCTACACTAGTGGAAGCGGAGCTACTAACTTCAGCCTGCTGAAGCAGGCTGGAGACGTGGAGGAG
AACCCTGGACCTTCTAGACTTAAGCTACTGTCTTCTATCGAACAAGCATGCGATATTTGCCGACTTAAAAAGCTCAAGTGCTCCAAAGAAAAACCGAAGTGCGCCAAGTGTCTGAAGAACAACTGGGAGTGTC
GCTACTCTCCCAAAACCAAAAGGTCTCCGCTGACTAGGGCACATCTGACAGAAGTGGAATCAAGGCTAGAAAGACTGGAACAGCTATTTCTACTGATTTTTCCTCGAGAAGACCTTGACATGATTTTGAAAAT
GGATTCTTTACAGGATATAAAAGCATTGTTAAGAGGATTATTTGTACAAGATAATGTGAATAAAGATGCCGTCACAGATAGATTGGCTTCAGTGGAGACTGATATGCCTCTAACATTGAGACAGCATAGAATA
AGTGCGACATCATCATCGGAAGAGAGTAGTAACAAAGGTCAAAGACAGTTGACTGTATCTTCGAGGTCGACCCCGGGAATTCAGATCTCTCGAGCCGCCCCCCCGACCGATGTCAGCCTGGGGGACGAGCTCC
ACTTAGACGGCGAGGACGTGGCGATGGCGCATGCCGACGCGCTAGACGATTTCGATCTGGACATGTTGGGGGACGGGGATTCCCCGGGTCCGGGATTTACCCCCCACGACTCCGCCCCCTACGGCGCTCTGGA
TATGTAGGCGGCCGCATTAAGTCTCAGCCACCGTTAACTGAACATGTCAAAACCTGTGGAGACTGTTGAGATTTGATGTTCTGAAAAGATAAAGCCTATAAATAAAATGTTGCCCAAATTTCCTGCCTGATGT
TTTTCTTTGTCTTTGCTACATGGCTTTGCTGCTCGGATCGGCTCACTCTGTGTATGCCACGTTCACTTTGTACTCTCCTTCTCACGGTAGGTTTATTATTTTTAGATGTGCAGTTAGTTTCTGTGAAATAACA
CACCACACACTGATATTGTCTGTGCATTGACTTGGTGAGTGCACATTGTTTTTGATCTTGACATATTTATATTTGATTGATCAGGTGAACTGTGTGAATCTAAAGTGCTCCATACAGATGTTCTGCATTGAAA
ATATTCTCATTTTATTAGTGGAAGTGAGTGTATGCCACATCCAATCAATTTCAGCAAACACCCCAGTATGATTTAATGCAAAAAAATGAAGGTATCAAACACGCATTACTACTTTGCAGTTAAATATTTAACA
TTTATTCCAACACGAAAAAAAGCAGTAAATAACACTTTGACAAACACGTCAGGACATCTTATTTTTGTCACCCTCACAGGCAATTTAGTATAATATATTATATATATATATATATCATATAATAATATTCAGT
ATAATATATATATATATATCATATTATAATATTCAGTATAATATAAAACACAAACACATATATGTATAATATAATATAACATTTTTATTTATTGAGATGCCTCTATGGACCGTGTTATAAGAAGTAAAGATCA
GGAGAAGTAAACATGAAGTGTAATTATGAATACTGATGTTAAATTAAGCTATGATGAGTTTTCACTGTTAATTTACCATCTCAATTAAATGTTGATGCCTCCATGACCAAGTTAAGCAGATGAGACTGAGACA
ACTGTAGAAGACAAGATGTTCACTTTGCTGAATATAGCTGGCTTGACAGTTATCTATGACTCTATAAATATATATATATATTTTTTTTTTTATAAAATGATTTATTTATAACTATATATCCATTTCTCAGACAGG
TGCTTCATATCCCTCACTCCCGTAGCTGTCCATGCTGGATCTGTCCCCGTTGTTTTTAAAAAGCTAAATAAGTTATTAACATGACTGCATCCAGCGAGCCAAACCTGTCTGGTGTACAGCTACCAGACCATGG
CGTTGTCTAGCAAGGAAGTGAAGAGCTATCCGGATATATATGTATGTTATCATAACTGTACTGGCTATGTCAGGTGGTAATGACTGTAACGTTACGTTACTCGTTGTAGGCACGACATTGAATATGCTCTTCT
CCGCTGTGGCCCGAACGCCTCCCGCTAGCGATGGGATCTCGGCCGCGATATCCCCTATAGTGAGTCGTATTACATGGTCATAGCTGTTTCCTGGCAGCTCTGGCCCGTGTCTCAAAATCTCTGATGTTACATT
GCACAAGATAAAAATATATCATCATGAACAATAAAACTGTCTGCTTACATAAACAGTAATACAAGGGGTGTTATGAGCCATATTCAACGGGAAACGTCGAGGCCGCGATTAAATTCCAACATGGATGCTGATT
TATATGGGTATAAATGGGCTCGCGATAATGTCGGGCAATCAGGTGCGACAATCTATCGCTTGTATGGGAAGCCCGATGCGCCAGAGTTGTTTCTGAAACATGGCAAAGGTAGCGTTGCCAATGATGTTACAGA
TGAGATGGTCAGACTAAACTGGCTGACGGAATTTATGCCTCTTCCGACCATCAAGCATTTTATCCGTACTCCTGATGATGCATGGTTACTCACCACTGCGATCCCCGGAAAAACAGCATTCCAGGTATTAGAA
GAATATCCTGATTCAGGTGAAAATATTGTTGATGCGCTGGCAGTGTTCCTGCGCCGGTTGCATTCGATTCCTGTTTGTAATTGTCCTTTTAACAGCGATCGCGTATTTCGTCTCGCTCAGGCGCAATCACGAA
TGAATAACGGTTTGGTTGATGCGAGTGATTTTGATGACGAGCGTAATGGCTGGCCTGTTGAACAAGTCTGGAAAGAAATGCATAAACTTTTGCCATTCTCACCGGATTCAGTCGTCACTCATGGTGATTTCTC
ACTTGATAACCTTATTTTTGACGAGGGGAAATTAATAGGTTGTATTGATGTTGGACGAGTCGGAATCGCAGACCGATACCAGGATCTTGCCATCCTATGGAACTGCCTCGGTGAGTTTTCTCCTTCATTACAG
AAACGGCTTTTTCAAAAATATGGTATTGATAATCCTGATATGAATAAATTGCAGTTTCATTTGATGCTCGATGAGTTTTTCTAATCAGAATTGGTTAATTGGTTGTAACACTGGCAGAGCATTACGCTGACTT
GACGGGACGGCGCAAGCTCATGACCAAAATCCCTTAACGTGAGTTACGCGTCGTTCCACTGAGCGTCAGACCCCGTAGAAAAGATCAAAGGATCTTCTTGAGATCCTTTTTTTCTGCGCGTAATCTGCTGCTT
GCAAACAAAAAAACCACCGCTACCAGCGGTGGTTTGTTTGCCGGATCAAGAGCTACCAACTCTTTTTCCGAAGGTAACTGGCTTCAGCAGAGCGCAGATACCAAATACTGTTCTTCTAGTGTAGCCGTAGTTA
GGCCACCACTTCAAGAACTCTGTAGCACCGCCTACATACCTCGCTCTGCTAATCCTGTTACCAGTGGCTGCTGCCAGTGGCGATAAGTCGTGTCTTACCGGGTTGGACTCAAGACGATAGTTACCGGATAAGG
CGCAGCGGTCGGGCTGAACGGGGGGTTCGTGCACACAGCCCAGCTTGGAGCGAACGACCTACACCGAACTGAGATACCTACAGCGTGAGCTATGAGAAAGCGCCACGCTTCCCGAAGGGAGAAAGGCGGACAG
GTATCCGGTAAGCGGCAGGGTCGGAACAGGAGAGCGCACGAGGGAGCTTCCAGGGGGAAACGCCTGGTATCTTTATAGTCCTGTCGGGTTTCGCCACCTCTGACTTGAGCGTCGATTTTTGTGATGCTCGTCA
GGGGGGCGGAGCCTATGGAAAAACGCCAGCAACGCGGCCTTTTTACGGTTCCTGGCCTTTTGCTGGCCTTTTGCTCACATGTT
```

Figure S1. Sequence of the donor plasmid. DNA sequence of the donor plasmid with gene-breaking cassette (3440 bp) before the insertions of the 5´and 3´homology arms.

SgRNA target site in intron 5 of *etv6*:

TGGGCGGGGATTTCCTTCTT<u>TGG</u>

PAM

Sequence 1:

```
GTGAGGCTCCACCCACAAGTGGGGCTAGCTTAGCCACTCCCCCACCTTCTATATAACTGAAACAGGCCTGATA
TTAGAAACAATGTGGGCGGGGATTTCCTTCTTTGGAAGCTGATTGGTTGAATGATGGGGGTTAACAAAATGAG
GGAAGATGGAGAAAAACAGGGCAGAAAAGAGACATTCATGGCCCAACACCAAATGACACGTAGCTTCGCCCTA
AGTAACTGATGGCCCCGCCCTAAATGACAAATATATCCGCCCTAAATGATGCATAGCTCCTCCTTAAATGACA
GATAGCCCTGTCTGGTAGATCCATCCTAAGTGACCAATATCTCCGCCCTAATTGACTGATAGATCCACCCTAA
```

Sequence 2:

```
GTGAGGCTCCACCCACAAGTGGGGCTAGCTTAGCCACTCCCCCACCTTCTATATAACTGAAACAGGCCTAATA
TTAGAAACAATGTGGGCGGGGATTTCCTTCTTCGGAATCTGATTGGTTGAATGATGGGGGTTAACAAAATGAG
GGAAGATGGAGAAAAACAGGGCAGAAAAGAGACATGCATGGCCCAACACCAAATGACACGTAGCTTCGCCCTA
AGTAACTGATAGCCCCGCCCTAAATGACAAATATATCCGCCCTAAATGATGCATAGCTCCTCCTTAAATGACA
GATAGCCCTGTCTGGTAGATCCATCCTAAGTGACCGATATCTCCGCCCTAATTGACTGATAGATCCACCCTAA
```

Figure S2. Sequences of intron 5 of *etv6*. Partial sequences from both *etv6* alleles of the *UAS:GFP* line were determined by Sanger sequencing. SgRNA target site is shown. PAM sequence is underlined.

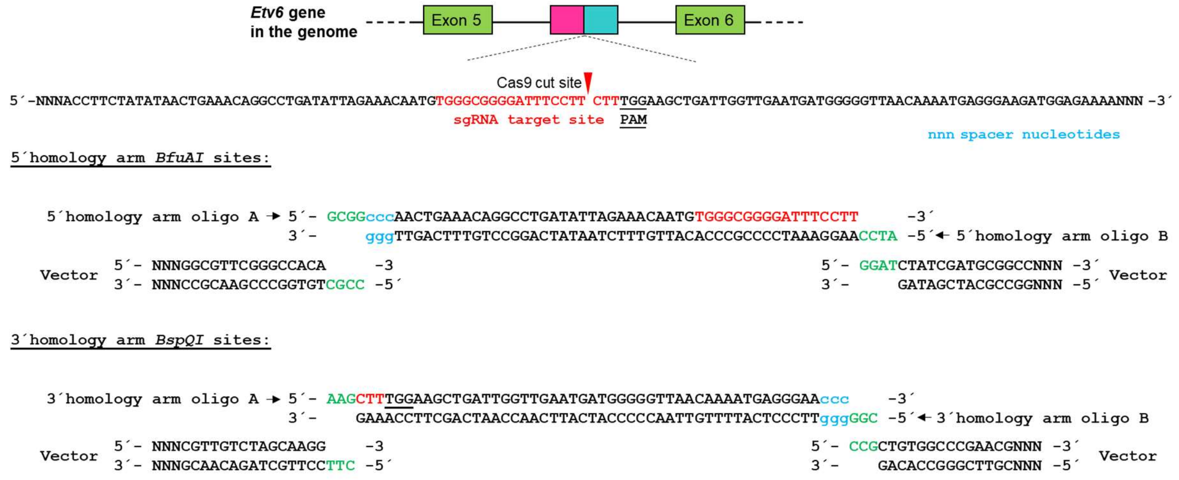

Figure S3. Homology arm design. Scheme of CRISPR/Cas9 target site in intron 5 of *etv6.* SgRNA sequence in red and PAM sequence underlined. Annealed 5´and 3´ homology arm oligos A and B are shown with overhangs (green) complementary to the overhangs after enzyme digestion in the donor plasmid. Pink and turquoise rectangles: homology arms (48 bp).

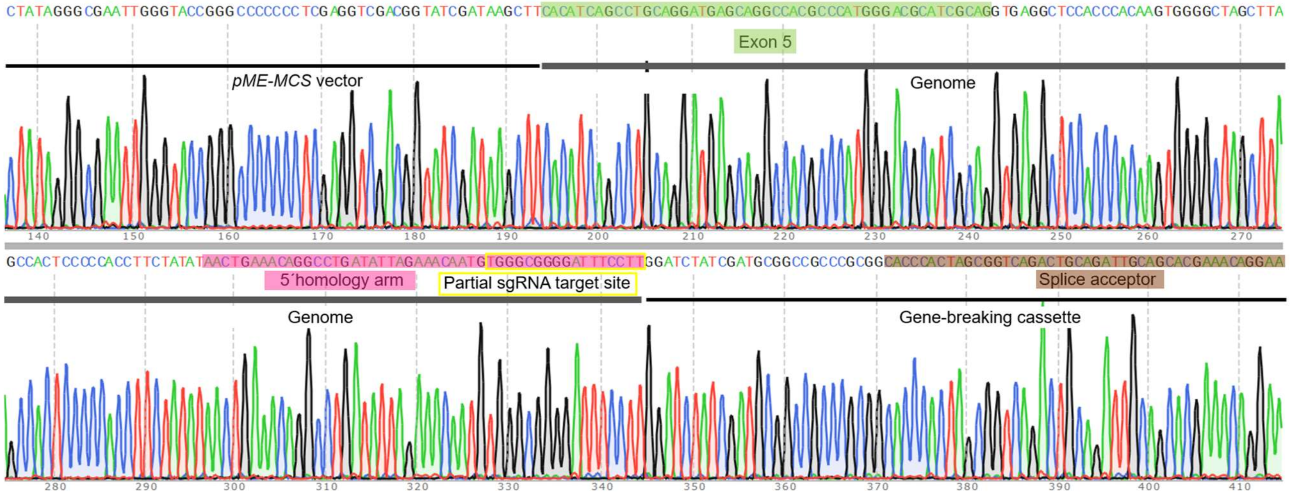

Figure S4. Chromatogram of Sanger sequencing of the genomic integration. The Sanger sequencing chromatogram revealed the precise integration of the gene-breaking cassette 5´ to the genomic double-strand break in the genome. A fragment of exon 5 of etv6 (green), the 5´ homology arm (pink), portions of the sgRNA target site (yellow frame), and a segment of the splice acceptor (brown) are displayed.

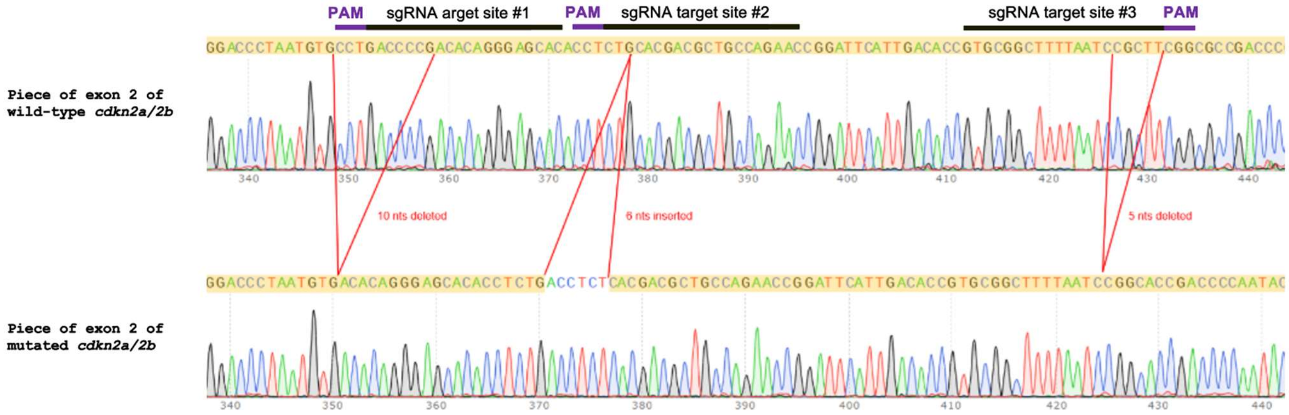

Figure S5. Sanger sequencing analysis of exon 2 of the *cdkn2a/b* gene in the *E::R;cdkn2a/b*$^{+/-}$ zebrafish line. The wild-type sequence and the mutated sequence are displayed. The three sgRNA target sites and PAM sites are indicated. In mutated exon 2, there are ten nucleotides deleted, six nucleotides inserted, and five nucleotides deleted. This results in a premature stop codon occurring after 210 nucleotides.

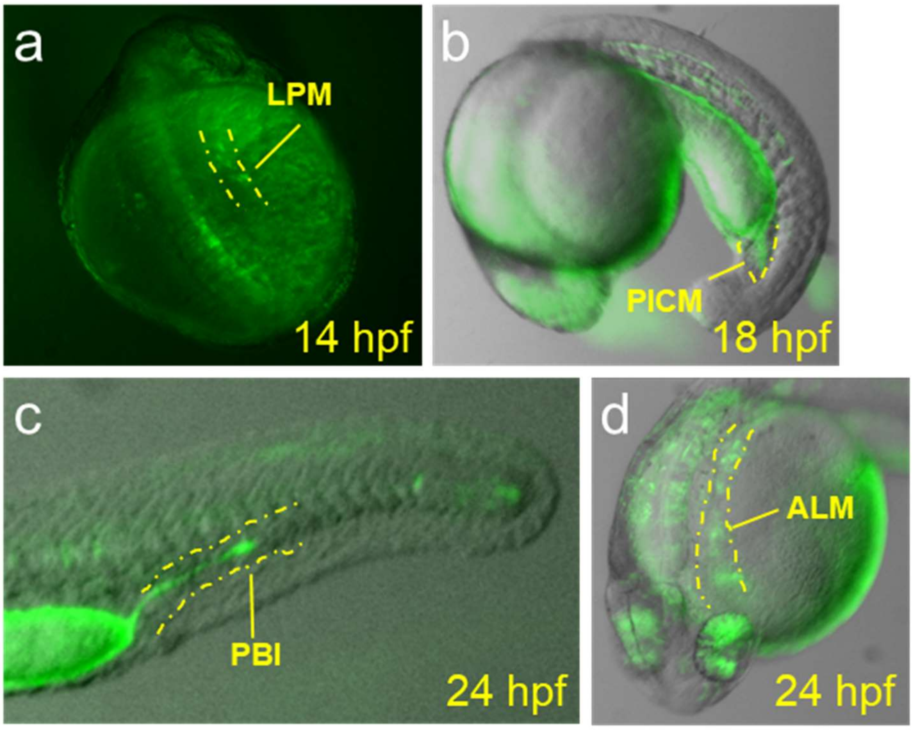

Figure S6. GFP Expression in *E::R* knock-in line during primitive hematopoiesis. (a) Dorsal view of the embryo with the anterior at the top. GFP-expressing cells are observed in the lateral plate mesoderm (LPM) at 14 hpf. (b) In a lateral view of the embryo with the anterior to the left, GFP-expressing cells are found in the posterior intermediate cell mass (PICM) at 18 hpf. (c) A close-up view of the caudal region of the embryo (anterior to the left) reveals GFP-expressing cells in the posterior blood island (PBI) at 24 hpf. (d) Dorsal view of the embryo with the anterior at the bottom shows GFP-expressing cells in the anterior lateral plate mesoderm (ALM) at 24 hpf. Hpf: hours post-fertilization.

(a)

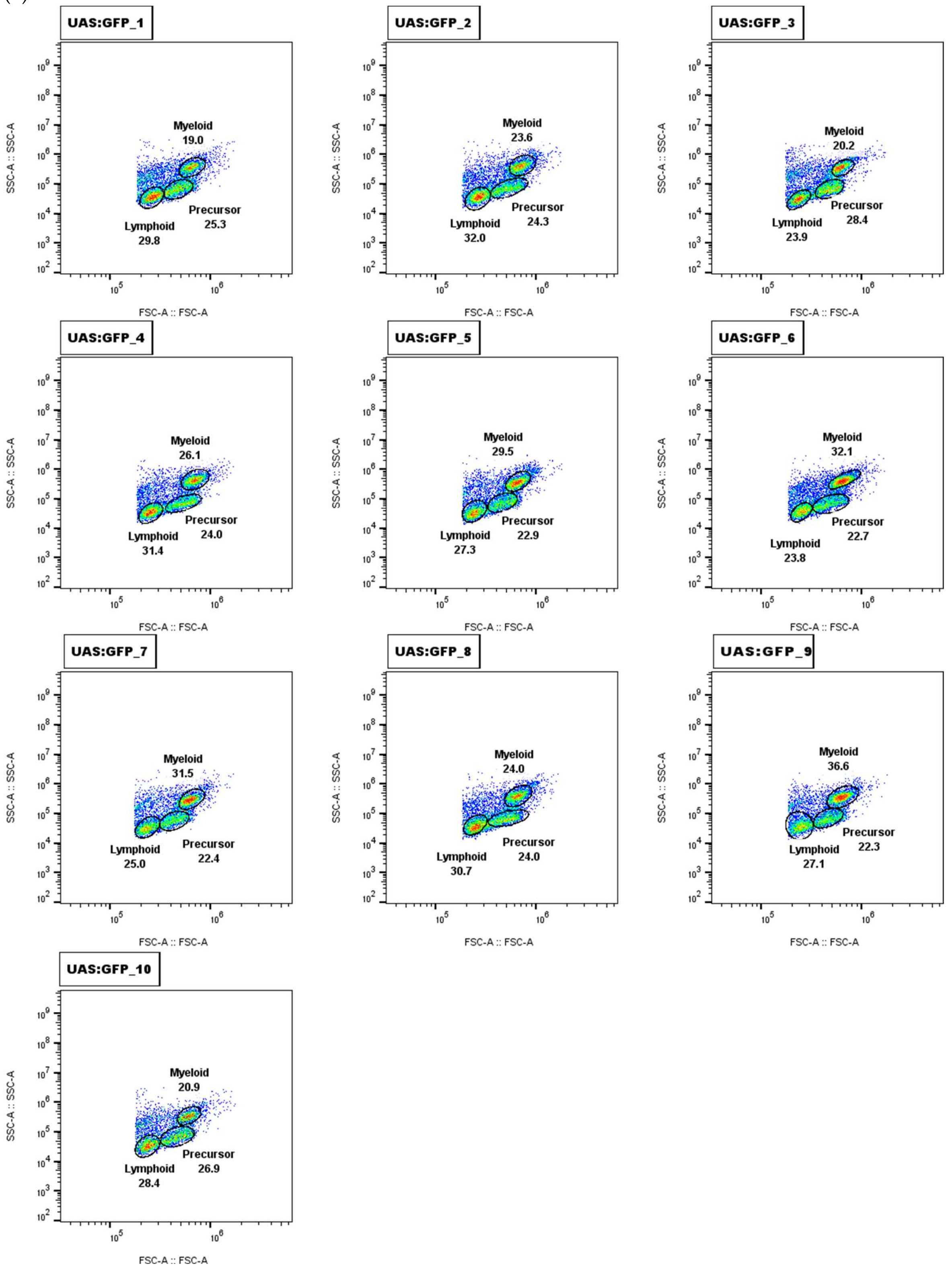
UAS:GFP_1
Myeloid 19.0
Precursor 25.3
Lymphoid 29.8
UAS:GFP_2
Myeloid 23.6
Precursor 24.3
Lymphoid 32.0
UAS:GFP_3
Myeloid 20.2
Precursor 28.4
Lymphoid 23.9
UAS:GFP_4
Myeloid 26.1
Precursor 24.0
Lymphoid 31.4
UAS:GFP_5
Myeloid 29.5
Precursor 22.9
Lymphoid 27.3
UAS:GFP_6
Myeloid 32.1
Precursor 22.7
Lymphoid 23.8
UAS:GFP_7
Myeloid 31.5
Precursor 22.4
Lymphoid 25.0
UAS:GFP_8
Myeloid 24.0
Precursor 24.0
Lymphoid 30.7
UAS:GFP_9
Myeloid 36.6
Precursor 22.3
Lymphoid 27.1
UAS:GFP_10
Myeloid 20.9
Precursor 26.9
Lymphoid 28.4
SSC-A :: SSC-A
FSC-A :: FSC-A

(b)

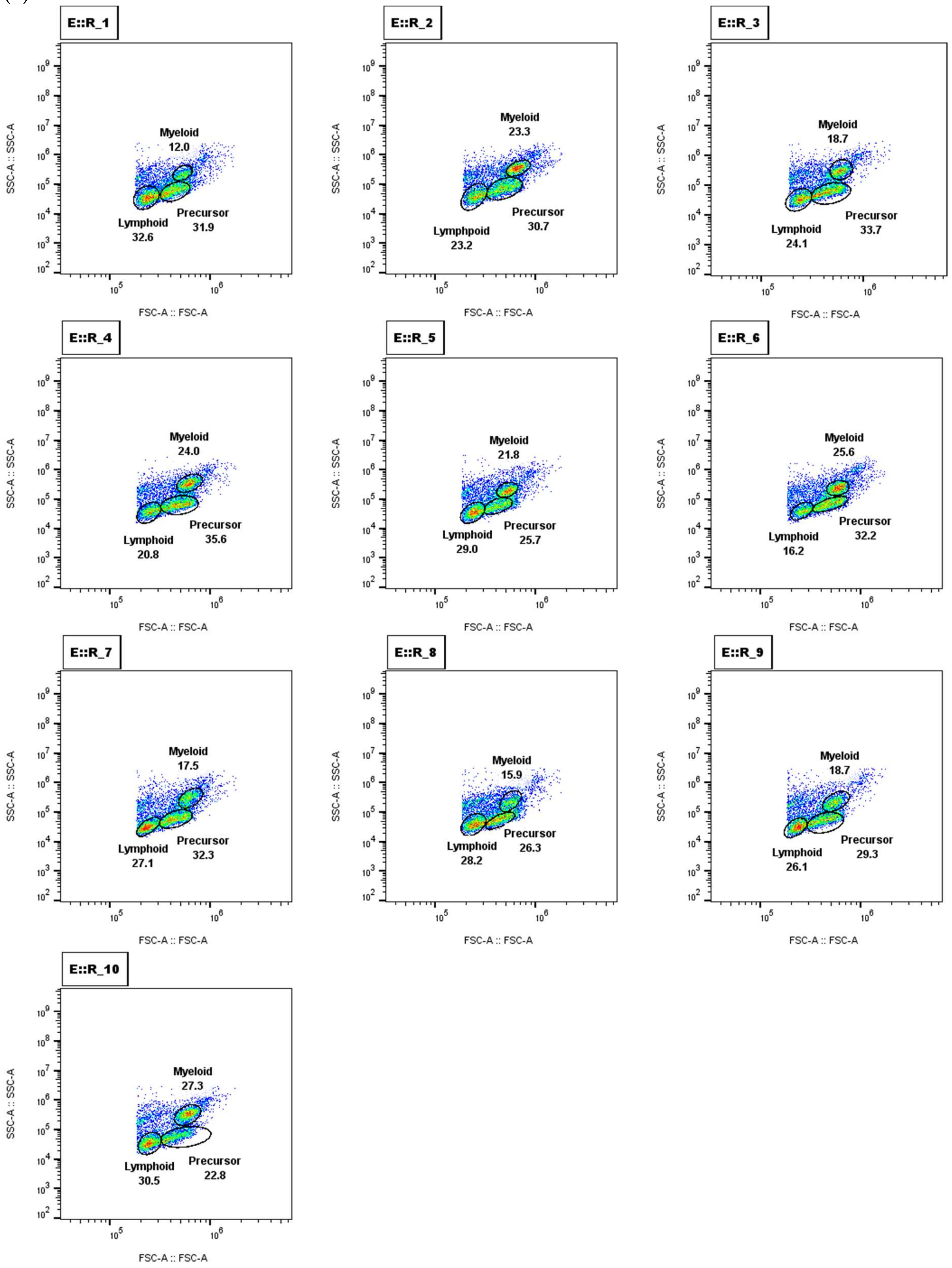

Figure S7. Flow cytometry plots. Flow cytometry plots depicting the lymphoid, precursor, and myeloid populations isolated from the whole kidney marrows of ten *UAS:GFP* zebrafish (a) and ten *E::R* zebrafish (b), categorized based on their light scatter characteristics.

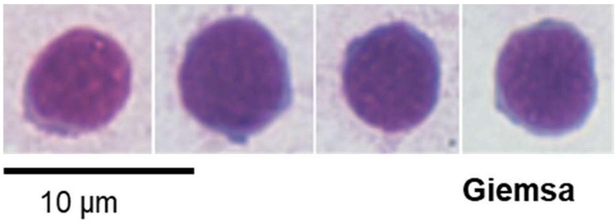

Figure S8. Giemsa staining of peripheral blood shows high nuclear-cytoplasmic ratio of the lymphoblasts in the *E::R* knock-in fish.

Figure 3d original blot.

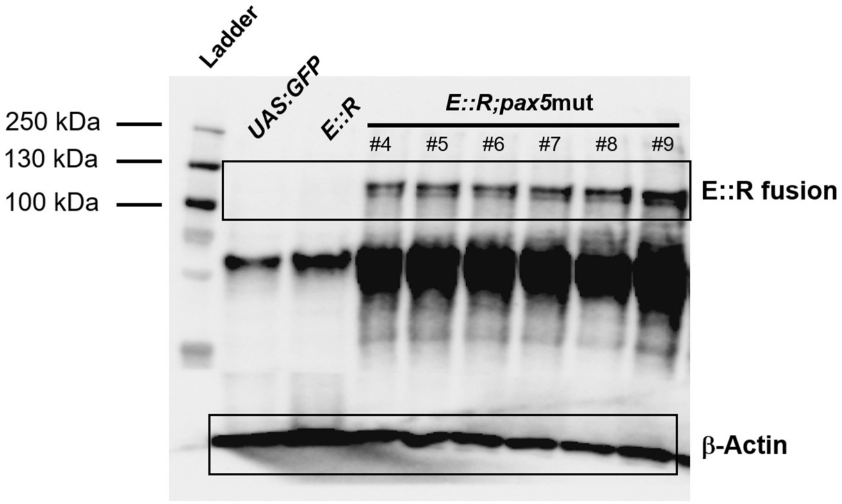

Figure S9. Uncropped Western blot of *UAS:GFP*, non-leukemic *E::R* and leukemic *E::R;pax5*mut zebrafish lines by using the human anti-RUNX1 antibody.

Tumor # 1; exon 3 of *pax5* contains either wild-type or an insertion of 2 bases (GC)

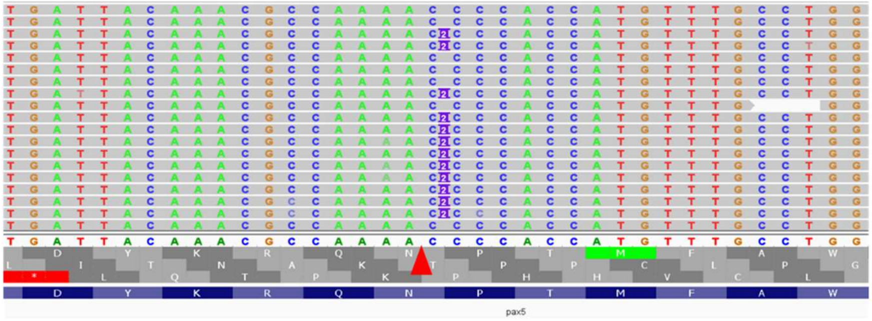

Genomic target of gRNA: 5´**AGGCAAACATGGTGGGGTTT** 3´ (located on the reverse strand)

PAM: **TGG**

▲ Cutting position

Tumor # 1; exon 5 of *pax5* contains either a deletion (4 bases: GCAG) or an insertion (13 bases: AAACATGGTAGGC)

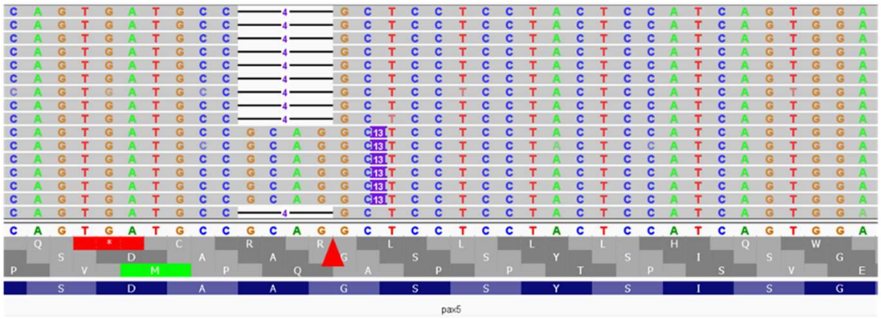

Genomic target of gRNA: 5´**GATGGAGTAGGAGGAGCCT** 3´ (located on the reverse strand)

PAM: **CGG**

▲ Cutting position

Tumor # 2; exon 3 of *pax5* contains either a deletion of 6 bases (CCAAAA) or a deletion of 4 bases (CCCC) followed by the exchange of three bases (A->T; C->G; C->A)

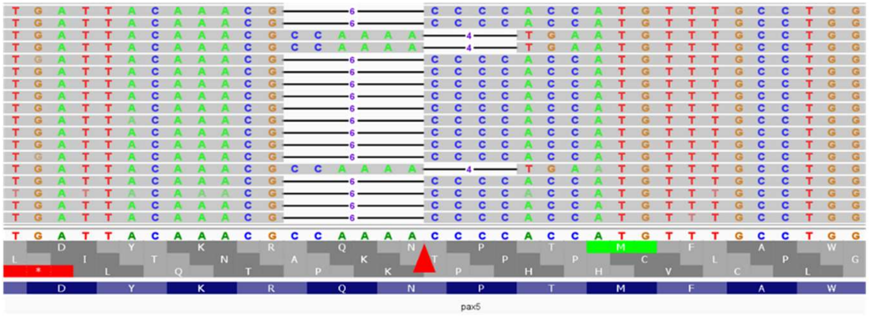

Genomic target of gRNA: 5´ **AGGCAAACATGGTGGGGTTT** 3´ (located on the reverse strand)

PAM: **TGG**

▲ Cutting position

Tumor # 2; exon 5 of *pax5* contains either a deletion (4 bases: GCAG) or an insertion (13 bases: AAACATG[illegible]

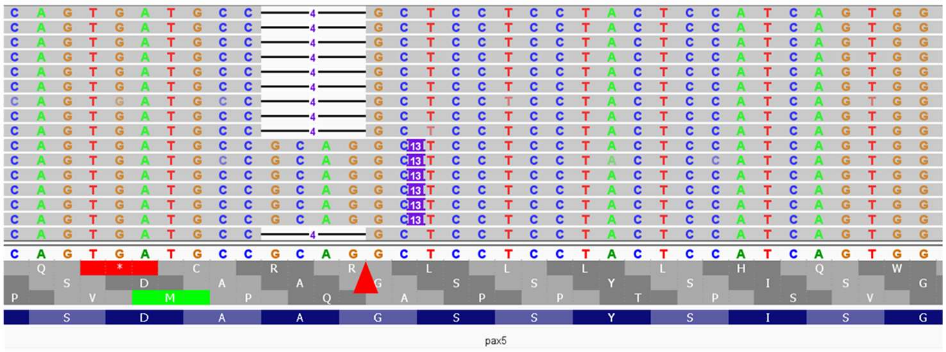

Genomic target of gRNA: 5´ **GATGGAGTAGGAGGAGCCT** 3´ (located on the reverse strand)

PAM: **CGG**

▲ Cutting position

Tumor # 3; exon 3 of *pax5* contains no mutations

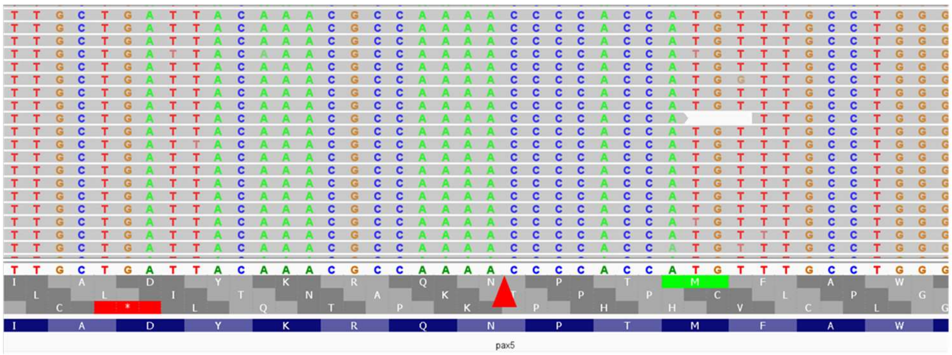

Genomic target of gRNA: 5´**AGGCAAACATGGTGGGGTTT** 3´ (located on the reverse strand)

PAM: **TGG**

▲ Cutting position

Tumor # 3; exon 5 of *pax5* contains either a deletion of 1 or 7 bases

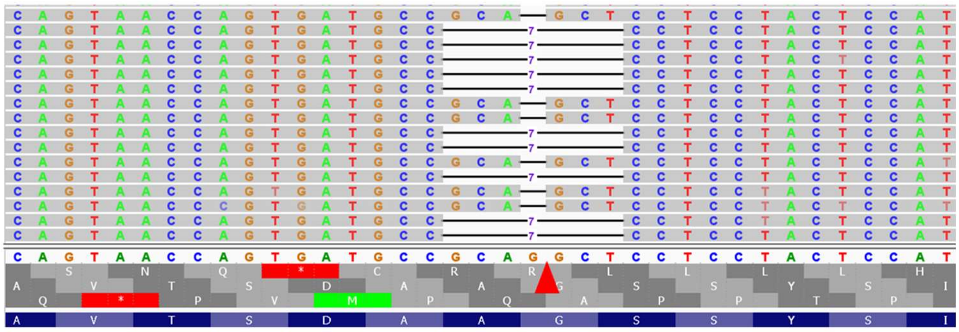

Genomic target of gRNA: 5´ **GATGGAGTAGGAGGAGCCT** 3´ (located on the reverse strand)

PAM: **CGG**

▲ Cutting position

Figure S10. Integrative Genomics Viewer screenshots illustrating mutations in *pax5* exon 3 and exon 5 in *E::R;pax5*mut zebrafish. Tumors #1-#3 are selected as representative examples.

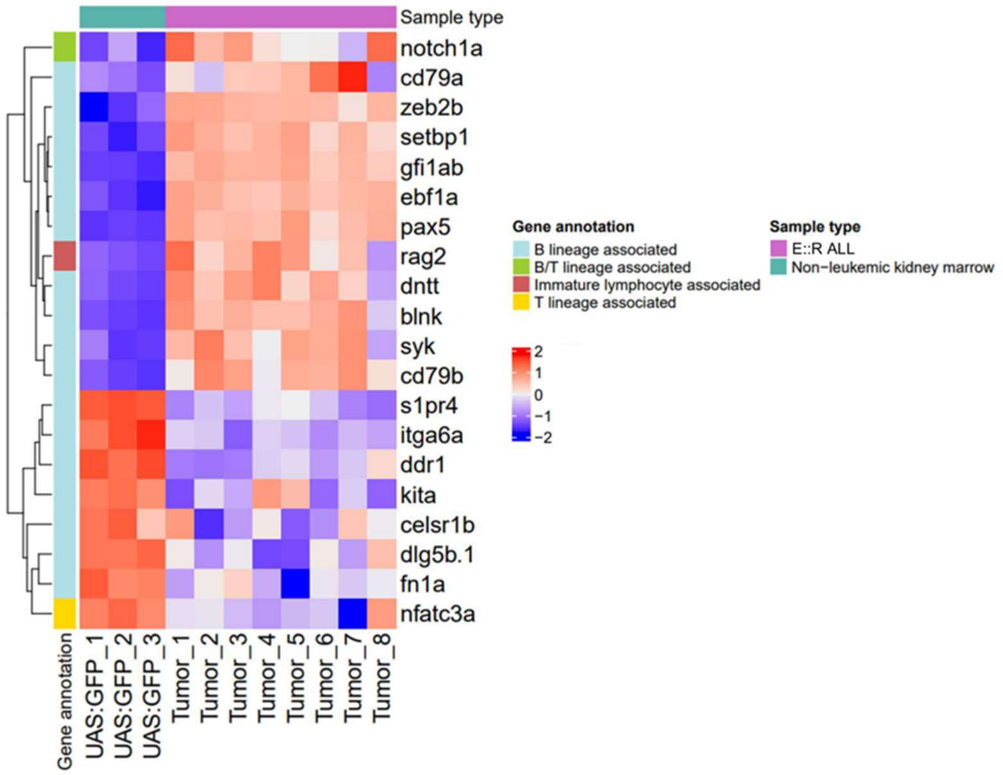

Figure S11. Heatmap visualizing the significantly differentially expressed (adjusted p-value ≤ 0.05) B and T lineage associated genes between the leukemic *E::R;pax5*mut and non-leukemic kidney marrow zebrafish transcriptomes, and their expression across the two groups.

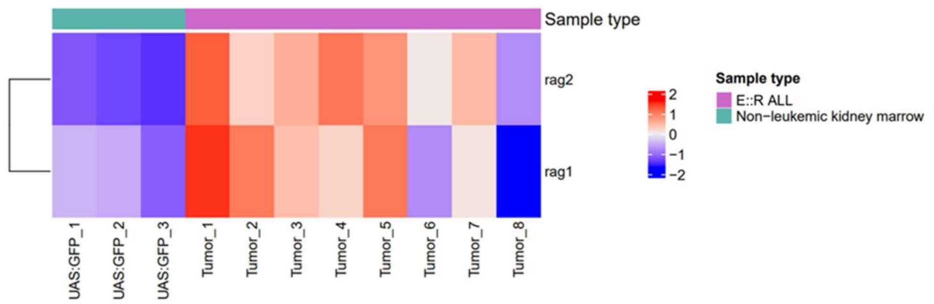

Figure S12. Heatmap visualizing the expression of *rag1* and *rag2* genes across the leukemic *E::R;pax5*mut and non-leukemic kidney marrow zebrafish transcriptomes.

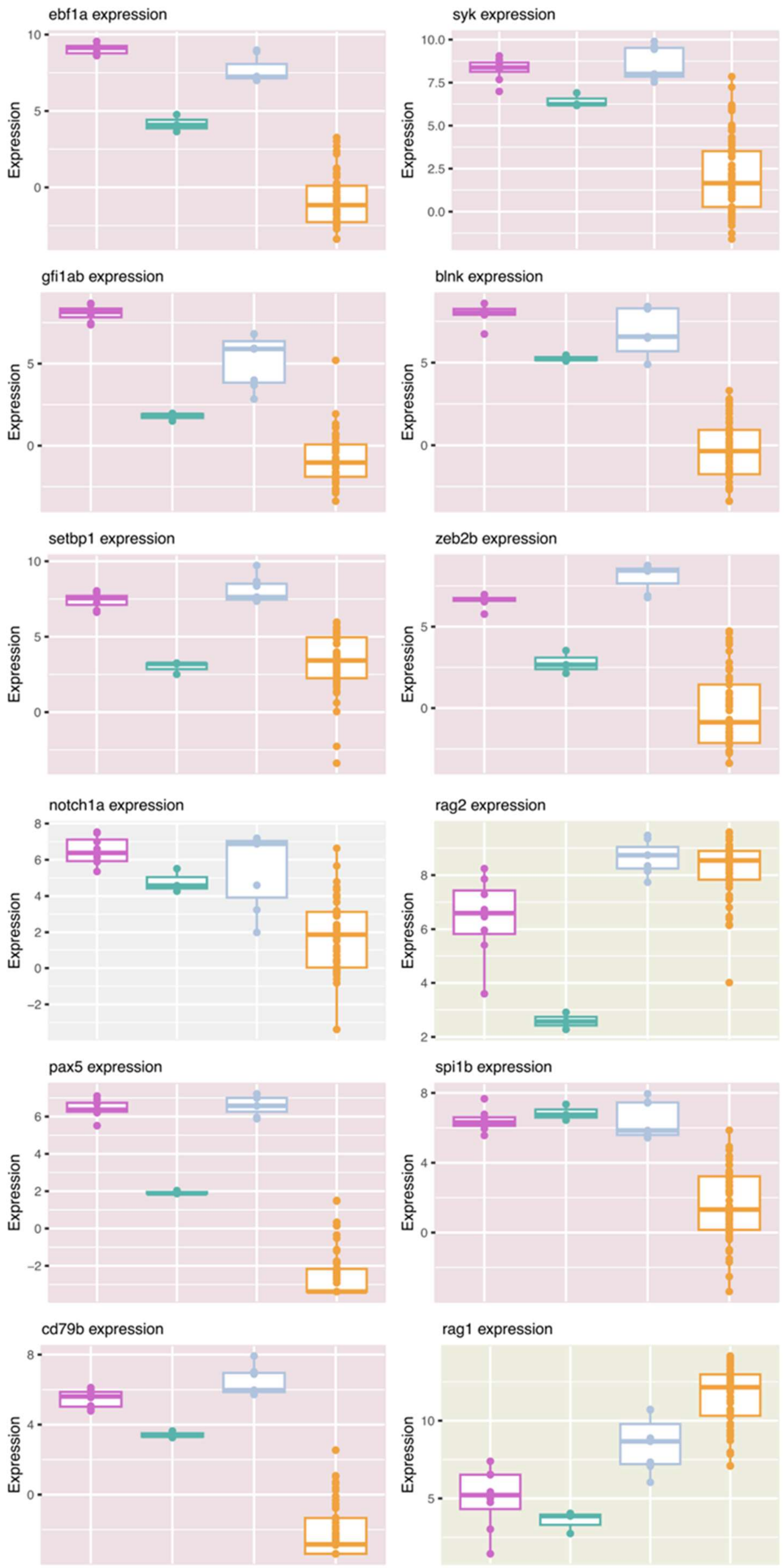
ebf1a expression
Expression
10
5
0
syk expression
Expression
10.0
7.5
5.0
2.5
0.0
gfi1ab expression
Expression
5
0
blnk expression
Expression
5
0
setbp1 expression
Expression
10
5
0
zeb2b expression
Expression
5
0
notch1a expression
Expression
8
6
4
2
0
−2
rag2 expression
Expression
8
6
4
2
pax5 expression
Expression
6
4
2
0
−2
spi1b expression
Expression
8
4
0
cd79b expression
Expression
8
4
0
rag1 expression
Expression
10
5

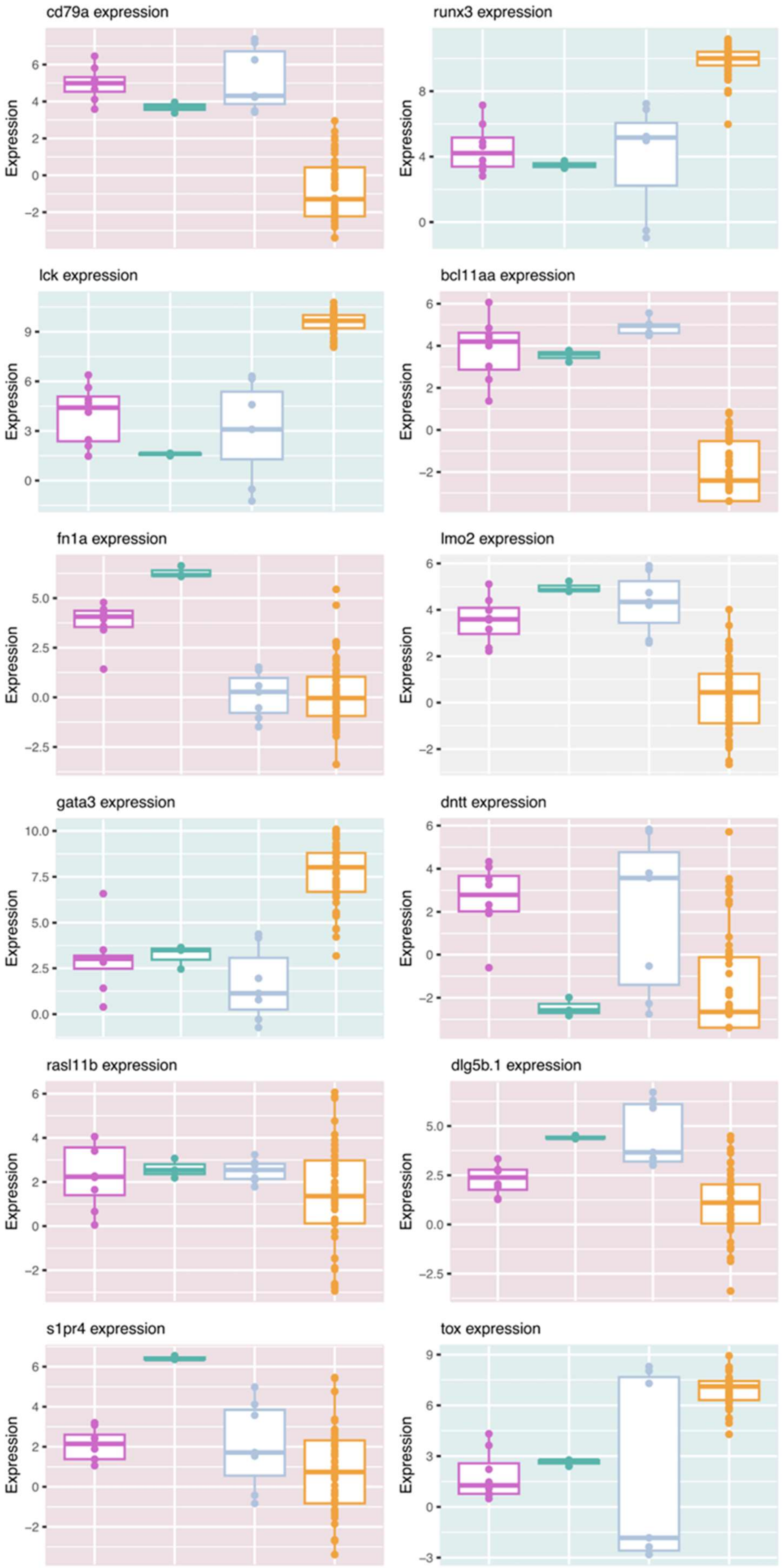

cd79a expression
runx3 expression
lck expression
bcl11aa expression
fn1a expression
lmo2 expression
gata3 expression
dntt expression
rasl11b expression
dlg5b.1 expression
s1pr4 expression
tox expression
Expression

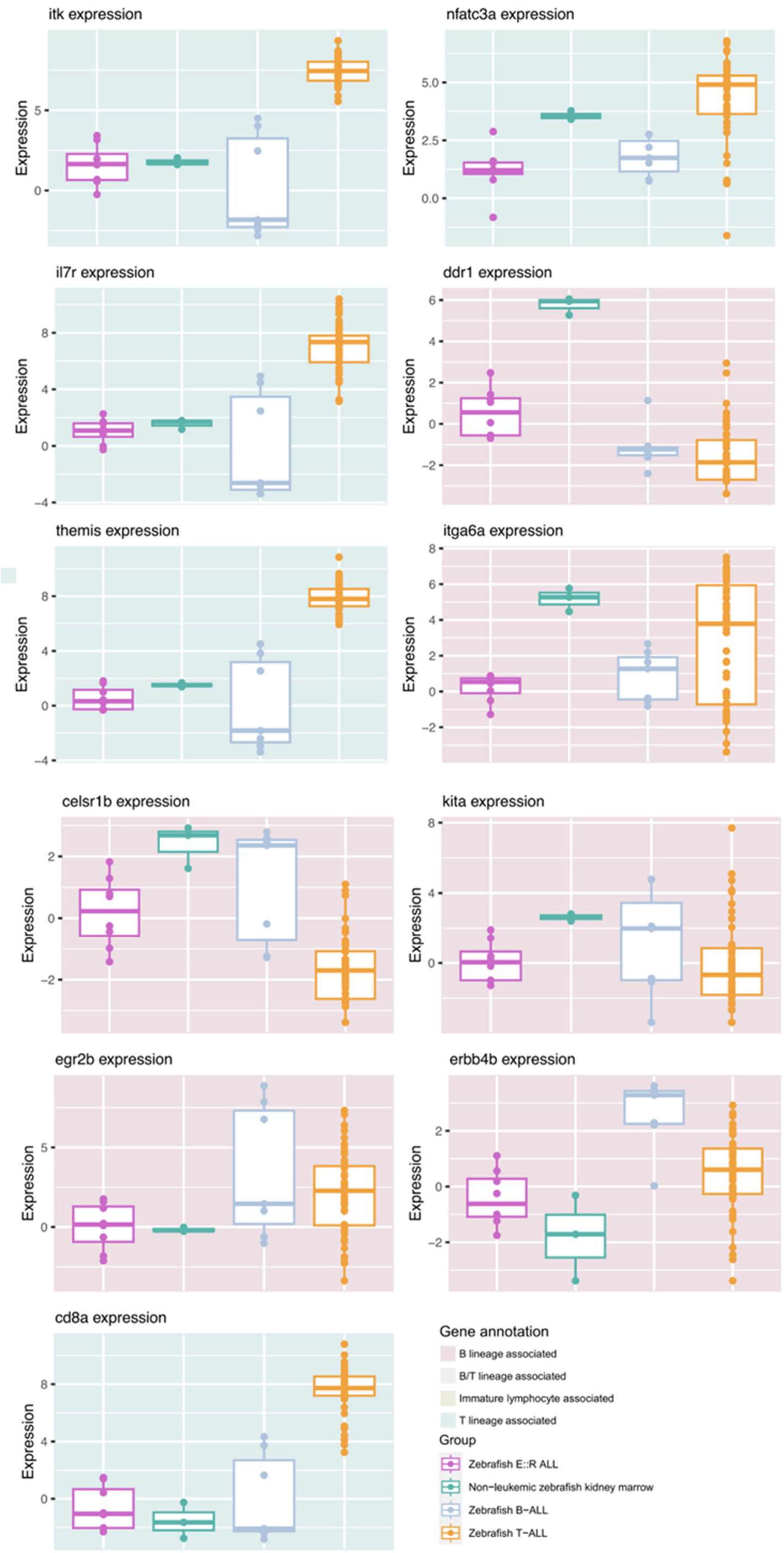

itk expression
nfatc3a expression
il7r expression
ddr1 expression
themis expression
itga6a expression
celsr1b expression
kita expression
egr2b expression
erbb4b expression
cd8a expression
Expression
Gene annotation
B lineage associated
B/T lineage associated
Immature lymphocyte associated
T lineage associated
Group
Zebrafish E::R ALL
Non-leukemic zebrafish kidney marrow
Zebrafish B-ALL
Zebrafish T-ALL

Figure S13. Boxplots describing the gene expression level of B and T lineage associated genes. between the zebrafish *E::R* ALLs (n=8), non-leukemic kidney marrow (n=3) B-ALLs (n=7), and T-ALLs (n=55). Expression values are log2-transformed counts per million values, and horizontal line corresponds to the median expression level in each group.

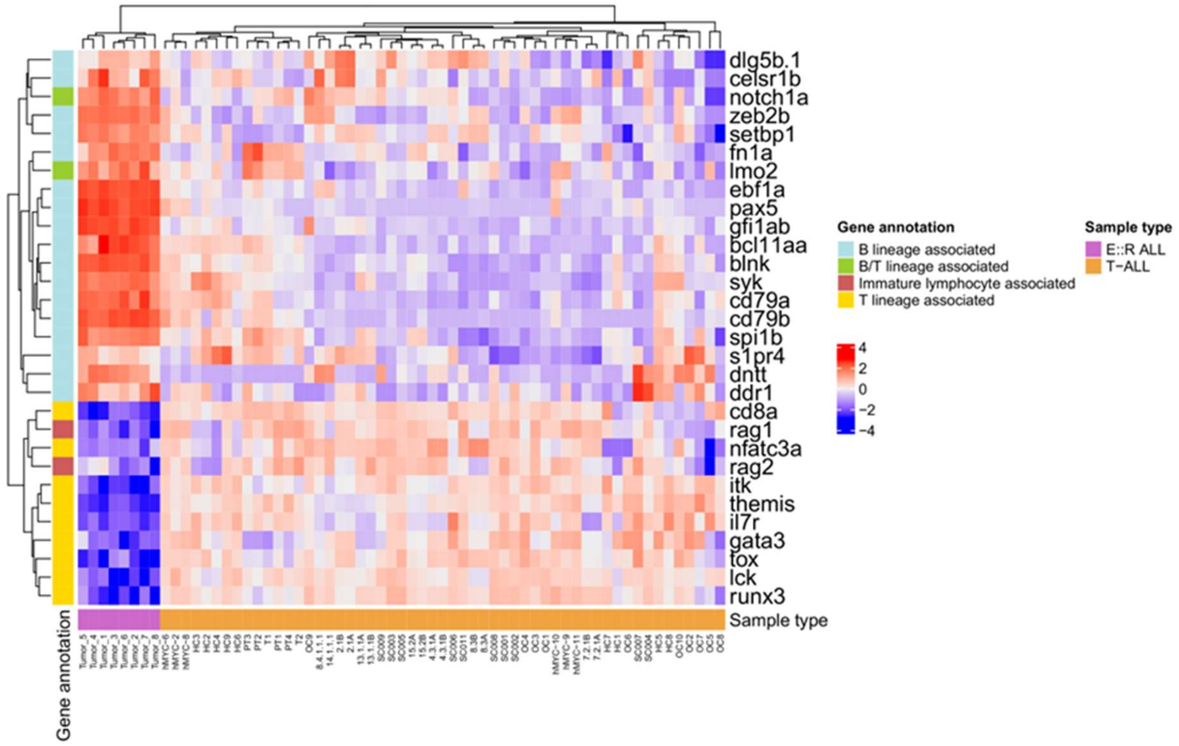

Figure S14. Heatmap visualizing the significantly differentially expressed (adjusted p-value ≤ 0.05) B and T lineage associated genes between the *E::R;pax5*mut ALL and T-ALL zebrafish transcriptomes, and their expression across the two different zebrafish leukemia types. *E::R;pax5*mut zebrafish leukemias express several B lineage associated genes at significantly higher levels compared to zebrafish with T-ALL, while the expression difference is the opposite for multiple T lineage associated genes.

Table S1. Oligos and templates used for the construction of the donor plasmid.

Purpose	Sequence (5´-> 3´)	Template
Destroying *XbaI* site	Top oligo: CTAGGTAGGGATAACAGGGTAATA	-
	Bottom oligo: AATTTATTACCCTGTTATCCCTAC	
Insertion of *ClaI-NotI-SacII-SpeI*-P2A-*XbaI-NcoI*-fragment	Top oligo: GATCTATCGATGCGGCCGCCCCGCGGACTAGTG GAAGCGGAGCTACTAACTTCAGCCTGCTGAAG CAGGCTGGAGACGTGGAGGAGAACCCTGGAC CTTCTAGAC	-
	Bottom oligo: CATGGTCTAGAAGGTCCAGGGTTCTCCTCCACG TCTCCAGCCTGCTTCAGCAGGCTGAAGTTAGTA GCTCCGCTTCCACTAGTCCGCGGGCGGCCGCAT CGATA	
Amplification of human *RUNX1* (exon 2-8)	Forward primer with *SacII*: AGACCGCGGAATGCATACTTG	*RUNX1* Human Untagged Clone in *pCMV6-XL5* (Origene; #SC123977)
	Reverse primer with *SpeI*: AGAACTAGTGTAGGGCCTCCACAC	
Amplification of carp b-actin intron 1 splice acceptor	Forward primer with *SacII*: AGACCGCGGCACCCACTAGCGGTC	*pGBT-RP2-1*
	Reverse primer with *XbaI*: AGATCTAGAAGGATTCATTCCAAGTATGCATTG CGGCTGAACTG	
Amplification of GAL4-VP16	Forward primer with *XbaI*: AGAGAATTCGTTAAGCTACTGTCTTC	*pME-Gal4VP16*
	Reverse primer with *NotI*: AGAGCGGCCGCCTACATATCCAGAGC	
Amplification of TE	Forward primer with *NotI*: AGAGCGGCCGCATTAAGTCTCAGCCAC	*pGBT-RP2-1*
	Reverse Primer with *NcoI*: AGACCATGGTCTGGTAGCTGTACAC	
Insertion of 5´ homology arm	Oligo A: GCGGCCCAACTGAAACAGGCCTGATATTAGAA ACAATGTGGGCGGGGATTTCCTT	-
	Oligo B: ATCCAAGGAAATCCCCGCCCACATTGTTTCTAA TATCAGGCCTGTTTCAGTTGGG	
Insertion of 3´ homology arm	Oligo A: AAGCTTTGGAAGCTGATTGGTTGAATGATGGG GGTTAACAAAATGAGGGAACCC	-
	Oligo B: CGGGGGTTCCCTCATTTTGTTAACCCCCATCATT CAACCAATCAGCTTCCAAAG	

Table S2. Targets of sgRNAs.

**Gene/plasmid** (GenBank number if applicable)	**Target sequence (5´-> 3´)**	**Strand**	**Targeted exon/intron**
**Donor plasmid**	GGGAGGCGTTCGGGCCACAG	-	-
Etv6 (BC045451.1)	TGGGCGGGGATTTCCTTCTT	Forward	Intron 5
Pax5 (BX511134.8)	AGGCAAACATGGTGGGGTTT	Reverse	Exon 3
	GATGGAGTAGGAGGAGCCTG	Reverse	Exon 5
Cdkn2a/2b (CT573245.20)	GTGCTCCCTGTGTCGGGGTC	Reverse	Exon 2
	GTTCTGGCAGCGTCGTGCAG	Reverse	Exon 2
	GTGCGGCTTTTAATCCGCTT	Forward	Exon 2

Table S3. Oligos for the generation of sgRNA templates.

**Oligo**	**Sequence (5´-> 3´)**
sgRNA intron 5 *etv6*	TAATACGACTCACTATAGGGGCGGGGATTTCCTTCTTGTTT TAGAGCTAGAA
sgRNA *pax5* exon 3	TAATACGACTCACTATAGGGCAAACATGGTGGGGTTTGTTT TAGAGCTAGAA
sgRNA *pax5* exon 5	TAATACGACTCACTATAGGTGGAGTAGGAGGAGCCTGGTT TTAGAGCTAGAA
sgRNA *cdkn2a/2b* exon 2 #1	TAATACGACTCACTATAGGGCTCCCTGTGTCGGGGTCGTTT TAGAGCTAGAA
sgRNA *cdkn2a/2b* exon 2 #2	TAATACGACTCACTATAGGTCTGGCAGCGTCGTGCAGGTTT TAGAGCTAGAA
sgRNA *cdkn2a/2b* exon 2 #1	TAATACGACTCACTATAGGGCGGCTTTTAATCCGCTTGTTT TAGAGCTAGAA
Universal oligo with guide RNA sequence	GATCCGCACCGACTCGGTGCCACTTTTTCAAGTTGATAAC GGACTAGCCTTATTTT AACTTGCTATTTCTAGCTCTAAAAC
T7 primer	TAATACGACTCACTATA
3´gRNA primer	GATCCGCACCGACTCGGTG

Table S4. Oligos used for T7 endonuclease I assays.

**Oligo**	**Sequence (5´-> 3´)**
Intron 5 of *etv6*	Forward: AGAGGATCCACAGACACTACCGCAAC
	Reverse: AGAAAGCTTGGTGGATCTATCAGTC
Pax5 exon 3	Forward: GTCTCGTGATATTCAGCCTTC
	Reverse: GAAGATGTGTTTTACACAC
Pax5 exon 5	Forward: GTAGTCTTATAGTCTCTCTG
	Reverse: CAAAGCATGCGCTGACTTAC
Cdkn2a/2b exon 2	Forward: AGAGGATCCGTGATGATGATGG
	Reverse: AGAAAGCTTTTAAACACGATTG

Table S5. Oligos used to amplify the genomic region over the insertion site of the gene-breaking cassette or a piece of the *E::R* cDNA.

Oligo	Sequence (5´-> 3´)
Forward primer with *HindIII*	AGAAAGCTTCACATCAGCCTGCAG
Reverse primer with *BamHI*	AGAGGATCCCATTCCAAGTATGCATT

Table S6. Significantly differentially expressed B and T lineage associated genes between the leukemic *E::R;pax5*mut and non-leukemic kidney marrow zebrafish transcriptomes and their adjusted p-values.

Gene name	Gene ID	Classification	P-value adjusted
syk	ENSDARG00000008186	B cell gene	5,25E-04
fn1a	ENSDARG00000019815	B cell gene	3,79E-03
pax5	ENSDARG00000037383	B cell gene	7,09E-06
cd79a	ENSDARG00000037473	B cell gene	0,02738069
dntt	ENSDARG00000038540	B cell gene	4,37E-02
itga6a	ENSDARG00000042282	B cell gene	7,84E-06
blnk	ENSDARG00000042722	B cell gene	0,0000209
kita	ENSDARG00000043317	B cell gene	3,02E-03
gfi1ab	ENSDARG00000044457	B cell gene	3,03E-06
rag2	ENSDARG00000052121	Immature lymphocyte gene	5,86E-03
celsr1b	ENSDARG00000058259	B cell gene	1,52E-02
s1pr4	ENSDARG00000074851	B cell gene	7,18E-06
nfatc3a	ENSDARG00000076297	T cell gene	2,88E-03
zeb2b	ENSDARG00000078416	B cell gene	6,43E-06
ddr1	ENSDARG00000078523	B cell gene	1,04E-05
dlg5b.1	ENSDARG00000090949	B cell gene	1,16E-03
setbp1	ENSDARG00000093799	B cell gene	5,38E-06
ebf1a	ENSDARG00000099849	B cell gene	9,54E-07
notch1a	ENSDARG00000103554	B cell gene / T cell gene	0,00350796
cd79b	ENSDARG00000104691	B cell gene	1,65E-04

Table S7. Significantly differentially expressed B and T lineage associated genes between *E::R* ALL, B-ALL, biphenotypic ALL, and T-ALL zebrafish transcriptomes and their adjusted p-values.

Gene name	Gene ID	Classification	P-value adjusted
spi1b	ENSDARG00000000767	B cell gene	1,10E-22
syk	ENSDARG00000008186	B cell gene	4,24E-25
themis	ENSDARG00000010619	T cell gene	6,84E-21
rasl11b	ENSDARG00000015611	B cell gene	0,020750432
gata3	ENSDARG00000016526	T cell gene	9,08E-15
itk	ENSDARG00000017565	T cell gene	5,82E-23
fn1a	ENSDARG00000019815	B cell gene	0,000241907
tox	ENSDARG00000032317	T cell gene	1,81E-16
pax5	ENSDARG00000037383	B cell gene	1,94E-50
cd79a	ENSDARG00000037473	B cell gene	2,15E-29
dntt	ENSDARG00000038540	B cell gene	2,08E-10
blnk	ENSDARG00000042722	B cell gene	2,63E-34
gfi1ab	ENSDARG00000044457	B cell gene	5,02E-36
cd8a	ENSDARG00000044797	T cell gene	1,11E-14
rag1	ENSDARG00000052122	Immature lymphocyte gene	1,66E-13
runx3	ENSDARG00000052826	T cell gene	2,86E-26
celsr1b	ENSDARG00000058259	B cell gene	1,96E-08
bcl11aa	ENSDARG00000061352	B cell gene	3,48E-30
s1pr4	ENSDARG00000074851	B cell gene	0,007295373
nfatc3a	ENSDARG00000076297	T cell gene	7,09E-06
zeb2b	ENSDARG00000078416	B cell gene	1,73E-33
ddr1	ENSDARG00000078523	B cell gene	0,020719094
il7r	ENSDARG00000078970	T cell gene	4,15E-12
dlg5b.1	ENSDARG00000090949	B cell gene	1,21E-07
setbp1	ENSDARG00000093799	B cell gene	1,20E-17
lmo2	ENSDARG00000095019	B cell gene / T cell gene	7,33E-19
ebf1a	ENSDARG00000099849	B cell gene	7,40E-42
lck	ENSDARG00000102525	T cell gene	3,13E-34
notch1a	ENSDARG00000103554	B cell gene / T cell gene	1,23E-16
cd79b	ENSDARG00000104691	B cell gene	1,55E-42

Table S8. Significantly differentially expressed B and T lineage associated genes between *E::R* ALL and T-ALL zebrafish transcriptomes and their adjusted p-values.

Gene name	Gene ID	Classification	P-value adjusted
spi1b	ENSDARG00000000767	B cell gene	1,99E-18
syk	ENSDARG00000008186	B cell gene	9,10E-20
themis	ENSDARG00000010619	T cell gene	2,67E-15
gata3	ENSDARG00000016526	T cell gene	2,65E-10
itk	ENSDARG00000017565	T cell gene	5,39E-18
fn1a	ENSDARG00000019815	B cell gene	6,54E-12
tox	ENSDARG00000032317	T cell gene	2,07E-11
pax5	ENSDARG00000037383	B cell gene	1,56E-44
cd79a	ENSDARG00000037473	B cell gene	1,90E-25
dntt	ENSDARG00000038540	B cell gene	3,77E-10
blnk	ENSDARG00000042722	B cell gene	6,44E-34
gfi1ab	ENSDARG00000044457	B cell gene	1,01E-49
cd8a	ENSDARG00000044797	T cell gene	6,55E-10
rag2	ENSDARG00000052121	Immature lymphocyte gene	0,000165878
rag1	ENSDARG00000052122	Immature lymphocyte gene	3,59E-14
runx3	ENSDARG00000052826	T cell gene	6,53E-19
celsr1b	ENSDARG00000058259	B cell gene	0,000175481
bcl11aa	ENSDARG00000061352	B cell gene	5,57E-24
s1pr4	ENSDARG00000074851	B cell gene	0,018002305
nfatc3a	ENSDARG00000076297	T cell gene	0,000202727
zeb2b	ENSDARG00000078416	B cell gene	5,67E-28
ddr1	ENSDARG00000078523	B cell gene	0,001689668
il7r	ENSDARG00000078970	T cell gene	8,95E-09
dlg5b.1	ENSDARG00000090949	B cell gene	0,011998089
setbp1	ENSDARG00000093799	B cell gene	6,23E-12
lmo2	ENSDARG00000095019	B cell gene / T cell gene	1,52E-13
ebf1a	ENSDARG00000099849	B cell gene	3,73E-42
lck	ENSDARG00000102525	T cell gene	4,57E-26
notch1a	ENSDARG00000103554	B cell gene / T cell gene	4,02E-16
cd79b	ENSDARG00000104691	B cell gene	4,12E-36

Table S9. Enriched KEGG pathways in *E::R;pax5*mut tumors (adjusted p-value ≤ 0.05). NumDEInCat: number of differential expressed genes in category; NumInCat: number of genes in category.

**KEGG pathway** (ID)	**NumDEInCat**	**NumInCat**	**P-value adjusted**
Spliceosome (03040)	96	126	2.5116e-11
Homologous recombination (03440)	21	24	0.0021
DNA replication (03030)	29	37	0.0021
Nucleotide excision repair (03420)	31	42	0.0059
Glycine, serine, and threonine metabolism (00260)	24	31	0.0118
ECM-receptor interaction (04512)	42	59	0.0150
Citrate cycle (00020)	25	33	0.0167
Mismatch repair (03430)	17	21	0.0198
Cell cycle (04110)	75	123	0.0205
Tryptophan metabolism (00380)	28	40	0.0305
Lysine degradation (00310)	30	43	0.0310
Pyrimidine metabolism (00240)	53	90	0.0310
Non-homologous end-joining (03450)	12	14	0.0310
RNA degradation (03018)	44	70	0.0310
Base excision repair (03410)	24	34	0.0310
Fatty acid degradation (00071)	26	37	0.0431
Metabolic pathways (01100)	492	989	0.0451
Histidine metabolism (00340)	17	23	0.0470

Table S10. Oligos used to amplify a piece of the *etv6* intron 5 via PCR.

**Oligo**	**Sequence (5´-> 3´)**
Forward primer with *BamHI*	AGAGGATCCACAGACACTACCGCAAC
Reverse primer with *HindIII*	AGAAAGCTTGGTGGATCTATCAGTC
