# Supplementary figures and images for: Generation of a Zebrafish Knock-In Model Recapitulating Childhood ETV6::RUNX1-Positive B-Cell Precursor Acute Lymphoblastic Leukemia
Source: Cancers (Basel). 2023 Dec 13;15(24):5821. doi: 10.3390/cancers15245821 (PMC10871125; doi:10.3390/cancers15245821)

Figure 3d original blot.

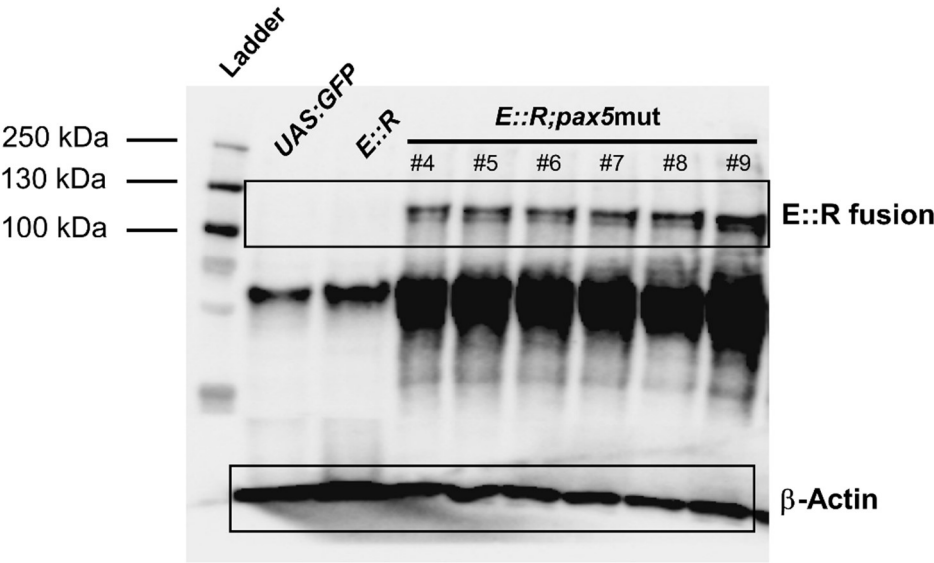

Supplement: Supplementary file 1 [file cancers-15-05821-s001.zip › cancers-2756528-original-images.pdf]
